# Experimentally-Based Computational Investigation into Beat-To-Beat Variability in Ventricular Repolarization and Its Response to Ionic Current Inhibition

**DOI:** 10.1371/journal.pone.0151461

**Published:** 2016-03-28

**Authors:** E. Pueyo, C. E. Dangerfield, O. J. Britton, L. Virág, K. Kistamás, N. Szentandrássy, N. Jost, A. Varró, P. P. Nánási, K. Burrage, B. Rodríguez

**Affiliations:** 1 Biomedical Research Networking Centre on Bioengineering, Biomaterials and Nanomedicine, University of Zaragoza, Zaragoza, Spain; 2 Biosignal Interpretation and Computational Simulation Group, I3A, IIS, Aragón, University of Zaragoza, Zaragoza, Spain; 3 Department of Computer Science, University of Oxford, Oxford, United Kingdom; 4 Department of Pharmacology and Pharmacotherapy, Faculty of Medicine, University of Szeged, Szeged, Hungary; 5 MTA-SZTE Research Group of Cardiovascular Pharmacology, Hungarian Academy of Sciences, Szeged, Hungary; 6 Department of Physiology, Faculty of Medicine, University of Debrecen, Debrecen, Hungary; 7 Department of Dental Physiology and Pharmacology, Faculty of Dentistry, University of Debrecen, Debrecen, Hungary; 8 School of Mathematical Sciences, Queensland University of Technology, Brisbane, Queensland, Australia; 9 ARC Centre of Excellence for Mathematical and Statistical Frontiers, Queensland University of Technology, Brisbane, Queensland, Australia; University of Milan, ITALY

## Abstract

Beat-to-beat variability in repolarization (BVR) has been proposed as an arrhythmic risk marker for disease and pharmacological action. The mechanisms are unclear but BVR is thought to be a cell level manifestation of ion channel stochasticity, modulated by cell-to-cell differences in ionic conductances. In this study, we describe the construction of an experimentally-calibrated set of stochastic cardiac cell models that captures both BVR and cell-to-cell differences in BVR displayed in isolated canine action potential measurements using pharmacological agents. Simulated and experimental ranges of BVR are compared in control and under pharmacological inhibition, and the key ionic currents determining BVR under physiological and pharmacological conditions are identified. Results show that the 4-aminopyridine-sensitive transient outward potassium current, I_to1_, is a fundamental driver of BVR in control and upon complete inhibition of the slow delayed rectifier potassium current, I_Ks_. In contrast, I_Ks_ and the L-type calcium current, I_CaL_, become the major contributors to BVR upon inhibition of the fast delayed rectifier potassium current, I_Kr_. This highlights both I_Ks_ and I_to1_ as key contributors to repolarization reserve. Partial correlation analysis identifies the distribution of I_to1_ channel numbers as an important independent determinant of the magnitude of BVR and drug-induced change in BVR in control and under pharmacological inhibition of ionic currents. Distributions in the number of I_Ks_ and I_CaL_ channels only become independent determinants of the magnitude of BVR upon complete inhibition of I_Kr_. These findings provide quantitative insights into the ionic causes of BVR as a marker for repolarization reserve, both under control condition and pharmacological inhibition.

## Introduction

As with many biological systems, variability in cardiac activity has been experimentally reported at a wide range of temporal and spatial scales from the molecular to the whole organ level [1], [2], [3], [4], [5], [6]. Variability in numbers of ion channels between different cells can lead to significant differences in the response to drugs or pathological conditions [7], [2], [3]. Furthermore, even the same cell exhibits beat-to-beat temporal electrophysiological variability, possibly caused by small random ionic current fluctuations arising from transitions between their channels’ states. In particular, marked differences in the duration of repolarization between consecutive action potentials (APs) are observed in single cell measurements, a phenomenon termed temporal beat-to-beat variability in repolarization duration (BVR) [8], [9].

BVR changes in response to pharmacological inhibition have been linked to the pro-arrhythmic potential of drug compounds, and elevated levels of BVR have been shown to successfully identify individuals at high risk of arrhythmia [10], [11], [12]. BVR quantified in isolated cardiomyocytes is substantially attenuated by gap junctional coupling in well-coupled tissue, and therefore its causal link with arrhythmic mechanisms may be limited in healthy tissue. However, BVR in isolated cells may represent a pro-arrhythmia indicator in conditions of reduced repolarization reserve caused by drugs, mutations or disease, and also impaired intercellular coupling, both known to enhance variability and pro-arrhythmic abnormalities in the heart [2], [13]. Understanding the ionic mechanisms underlying BVR in isolated cells may therefore help to inform its use as an arrhythmic risk biomarker (for example for drug testing), and also to better understand its causal relationship with arrhythmia in conjunction with other mechanisms [14], [15].

Computational studies using cardiac AP models with representation of stochasticity in one or several currents have contributed to the investigation of the role of stochastic ion channel dynamics in BVR [2], [16], [17]. All studies have highlighted the importance of cell-to-cell differences in the numbers of channels, and overall current conductances, as a key factor in modulating BVR. Cell-to-cell differences in conductances have, however, been mostly implemented in previous publications by sampling from specific statistical distributions rather than by using experimental calibration [2], [16], [17]. Thus, Pueyo et al. used truncated Gaussian distributions to construct guinea pig and human ventricular models incorporating the stochastic behaviour of the slow delayed rectifier potassium current (I_Ks_) based on voltage clamp and AP experimental recordings [2]. In the studies by Lemay et al. and Heijman et al., stochasticity was implemented in several currents using Poisson and Gaussian distributions, respectively, with no additional experimental input [16], [17]. The comparison of simulation and experimental results was therefore limited. The study by Lemay et al. was purely theoretical, whereas the latest comprehensive study by Heijman et al. provided a limited simulation/experimental evaluation based on the comparison of a single BVR measure versus AP duration (APD). Therefore, even though previous studies provide very valuable insights into potential determinants of BVR, none of them performed an experimental calibration step to select models that additionally satisfy physiological ranges in terms of AP and voltage clamp measurements from well-defined experimental datasets.

In this paper, we aim at quantifying the contribution of ion channel stochasticity to BVR in isolated canine ventricular myocytes in control and under pharmacological inhibition of various ion currents. To do so, we describe the construction and experimental calibration of a set of stochastic cardiac AP models consistent with AP and voltage clamp measurements. Our modelling approach considers the simultaneous variation of maximal conductances and ion channel numbers to assess the importance of cell-to-cell variations in ion channel numbers in the modulation of BVR. We specifically focus our investigations on the role on BVR of the main four ionic currents active during the AP repolarization phase, most of which are known to be key determinants of repolarization reserve [2], [4], [16], [17], [18]. These are the fast delayed rectifier potassium (K^+^) current, I_Kr_, the slow delayed rectifier K^+^ current, I_Ks_, the 4-aminopyridine-sensitive transient outward K^+^ current, I_to1_, and the L-type calcium (Ca^2+^) current, I_CaL_.

## Methods

### Experimental methods

#### Ethical Approval

All experimental data were obtained from single isolated canine ventricular cells, which were isolated for the purpose of this study. The experimental protocol conformed to the principles outlined in the Declaration of Helsinki and was approved by the local ethical committee (license N°: 18/2012/DEMÁB) and by the Department of Animal Health and Food Control of the Ministry of Agriculture and Rural Development, Hungary (XIII./1211/2012). Animals were only used as organ donors. Before the removal of their hearts, dogs were anaesthetized with intramuscular injections of 10 mg/kg ketamine hydrochloride (Calypsol, Richter Gedeon, Budapest, Hungary) and 1 mg/kg xylazine hydrochloride (Sedaxylan, Eurovet Animal Health BV, Bladel, The Netherlands).

Dogs were provided by an animal breeder named Feketerét Kutyakennel which is licenced by the appropriate Hungarian authority (Hajdú-Bihar Megyei Kormányhivatal, Élelmiszerlánc-biztonsági és Állategészségügyi Igazgatósága) under the number: II-KÁT/2015 to breed dogs for the purpose of scientific research. The breeder brought the dogs on the day of the experiment to the University of Debrecen, Faculty of Medicine, Department of Physiology, licenced by Hajdú-Bihar Megyei Kormányhivatal, Élelmiszerlánc-biztonsági, Növény és Talajvédelmi Főosztály as an experimental animal user facility under the number: III/3-KÁFH/2015.

According to the breeder, animals were allowed to have unrestricted amount of tap water to drink and food was given to them once a day in the evening except for the newborns and young ones to which food was given twice a day (morning and evening). The food was a mixture of cooked pork meat and bread factory waste where the proportion of the two components was designed according to the age of the animal. The dogs used in the experiments received only a minimal amount of food on the evening of the day before the experiment. Animals were housed in kennels. The minimum area of these kennels is 4 m2 for the first two animal + 2 m2 per each additional one. The height of the kennel is at least 2 m. Animals were kept in a flock where the number of animals was between 3 and 5. No surgery was performed on the animals.

#### Cell isolation

Single canine ventricular cells were acquired from adult beagle dog hearts by enzymatic dispersion using the segment perfusion technique as described in [19].

#### Voltage clamp recordings

Voltage clamp recordings were obtained for each of the repolarization currents using cardiomyocytes superfused with a Tyrode solution at 37°C. The composition of the Tyrode solution was (in mM): NaCl 144, NaH_2_PO_4_ 0.33, KCl 4.0, CaCl_2_ 1.8, MgCl_2_ 0.53, glucose 5.5, and HEPES 5.0. Suction pipettes were fabricated from borosilicate glass and had a tip resistance of 2 MΩ after filling with pipette solution containing (all in mM), K-aspartate 100, KCl 45, K-ATP 5, MgCl_2_ 1, EGTA 10, and HEPES 5 (pH 7.2) for measuring potassium currents or alternatively KCl 110, KOH 40, EGTA 10, HEPES 10, TEACl 20, MgATP 5, GTP 0.25 (pH was adjusted to 7.2 with KOH) for measuring calcium currents. To measure the potassium currents, 1 μM nisoldipine was added to the Tyrode solution in order to completely inhibit the inward calcium current (I_CaL_). For measurements of I_CaL_, 3 mM 4-aminopyridine was added to the Tyrode solution to eliminate the transient outward potassium current. Membrane currents (I_Kr_, I_Ks_, I_CaL_ and I_to1_) were recorded with the Axopatch-200B amplifier using the whole cell configuration of the patch clamp technique [20]. After establishing a high (1–10 GΩ) resistance seal by gentle suction, the cell membrane beneath the tip of the electrode was disrupted by further suction or by applying 1.5 V electrical pulses for 1 ms. The series resistance was typically 4–8 MΩ before compensation (usually 50–80%) and experiments were discarded when the series resistance was high or substantially increasing during the measurement. A 1000 Hz low pass filtering (Bessel filter, 80 dB/decade) was applied to the analogue current signals before the outputs from the amplifier were digitized at 100 kHz under software control (pClamp 6.0, Axon Instruments Inc.). A total of seven traces of each current type were considered for analysis in the present study. The results were analysed using software programs from Axon (pClamp 6.0 and 7.0, Axon Instruments, Foster City CA, USA).

#### Action potential recordings

Only rod-shaped viable cells showing clear striations were used and all electrophysiological measurements were performed at 37°C. Cells were sedimented in a plexiglass chamber and continuously superfused with modified Krebs solution composed of (in mM): NaCl, 128.3; NaHCO_3_, 21.4; KCl, 4.0; CaCl_2_, 1.8; MgCl_2_, 0.42; and glucose 10, gassed with a mixture of 95% O_2_ and 5% CO_2_ at pH = 7.4. Transmembrane potentials were recorded using 3 M KCl filled sharp glass microelectrodes with tip resistance between 20 and 40 MΩ and were connected to the input of either an Axoclamp-2B amplifier or a Multiclamp 700A amplifier (Axon Instruments Inc., Foster City, CA, USA). Cells were paced through the recording electrode at a steady cycle length of 1000 ms using 1–2 ms wide rectangular current pulses with 120% threshold amplitude. Large time-dependent changes were not observed in the APD for at least 60 minutes under these experimental conditions since the cytosol was not dialysed [19].

Before drug application, APs were recorded for 5 minutes to allow the cells to reach equilibrium. If AP parameters remained stable during this period the experiment was continued, otherwise it was aborted. After this initial period 50 consecutive APs were recorded with a basic cycle length (BCL) of 1000 ms, of which the last 30 beats were used for evaluation of BVR measures. The drug was applied after the control measurements were recorded and an incubation period of 5–6 minutes was allowed, which was adequate to obtain the steady-state drug effect. 50 consecutive APs were then recorded with a BCL of 1000 ms and again the last 30 of them were used for subsequent analysis. The following drug concentrations were used, along with the estimated channel inhibition: 0.1 μM of dofetilide which completely inhibited I_Kr_, 0.5 μM HMR-1556 which completely blocked I_Ks_ and 1 μM nisoldipine which gives 95% inhibition of I_CaL_ channel. The effects of double channel inhibition were also investigated using 0.5 μM HMR-1556 plus additional 100 μM Chromanol 293B which gives complete I_Ks_ inhibition and 90% I_to1_ inhibition. After measurements were taken for the initial application of HMR-1556, Chromanol 293B was applied and again allowed to obtain the steady-state drug effect before 50 consecutive AP measurements were taken. A total of 21 cells isolated from 7 hearts were used for evaluation of control conditions. For I_Kr_, I_Ks_, I_CaL_ and simultaneous I_Ks_ and I_to1_ inhibition the number of experiments were 5/4, 9/6, 7/5 and 7/3, respectively, meaning the number of cells/number of animals, with corresponding control cases for each inhibition condition. It is important to note that the experimental data were used to provide physiologically plausible ranges of biomarker values so as to calibrate the computational models in line with previous studies [2], [3]. Therefore, experiments were not used to investigate statistically significant differences in measurements or to explore potential ionic mechanisms of BVR, which would require additional measurements to be obtained. Furthermore, this number of experiments is in line with previous experimental studies [14]. The majority of cells used in this study are from the midmyocardial region, although the I_CaL_ inhibition recordings also contain subepicardial cells and the I_Kr_, I_Ks_ and I_Ks_ plus I_to1_ inhibition recordings include subepicardial and subendocardial cells as well.

### Computational methods

#### Cell Model

The canine epicardial model by Decker et al. [21], which is the most up-to-date electrophysiological model of the AP in a canine ventricular myocyte, provided the basic model structure for the set of models. The Decker model is an extension of the earlier Hund-Rudy model [22] and incorporates updated versions of the I_to1_, I_Ks_, I_CaL_ and sodium-potassium pump currents. We altered the steady-state activation parameter for I_CaL_ to better match the simulated morphology of the AP with available experimental data when I_CaL_ undergoes pharmacological inhibition:

$${ACT}_{\infty }=\frac{1}{\left(1+exp(\frac{-(V-15.3356)}{10.7558}))(1+exp(\frac{-(V+12.8824)}{2.1957})\right)}.$$

#### Construction of the experimentally-calibrated deterministic models

In order to take into consideration cell-to-cell variability in ionic conductances, we first constructed a deterministic set of models consisting of an ensemble of 1000 models sharing the same equations as the Decker model but with different values for the maximal conductances of I_Kr_, I_Ks_, I_to1_, I_CaL_ and I_K1,_ varied within ±100% of their original values by multiplying each conductance by a scaling factor (ranging from 0 to 2) generated using a Latin Hypercube sampling approach [3]. The number of models initially generated aimed at obtaining coverage of a wide range of the potential parameter space but without resulting in an exceedingly large number of models that could make the stochastic simulations intractable.

The next step consisted of the calibration of the set of deterministic models. We initially restricted the scaling factors applied to maximal ionic current conductances based on available voltage clamp experiments. Specifically, we computed the mean and standard deviation of normalized maximal current values and we allowed the scaling factors for the set of models to be up to 2.35 standard deviations from the mean of the experimental sample (as this would cover 98% of the observations if these followed a Gaussian distribution). We subsequently identified the models yielding APD values within experimental range under control conditions and following pharmacological inhibition. The pharmacological inhibition conditions were: complete I_Kr_ inhibition, complete I_Ks_ inhibition, 95% inhibition of I_CaL_ and complete inhibition of I_Ks_ combined with 90% inhibition of I_to1_. For each model, we calculated (1) APD (at 90% repolarization) for control conditions and pharmacological inhibition, and (2) change in APD due to inhibition of the currents (ΔAPD = APD^in drug^—APD^control^), averaged over five beats after steady-state pacing at 1 Hz. Only the models yielding APD and ΔAPD values up to 2.35 standard deviations from the mean of the experimental samples for control and all types of pharmacological inhibition were retained within the calibrated set of models.

#### Construction of the set of stochastic models

In all models within the calibrated set of deterministic models, the equations for I_Ks_, I_Kr_, I_to1_, and I_CaL_ were modified to include stochastic fluctuations using a reflected stochastic differential equation (SDE) taking the general form [23]:

$$dX_{t}=AX_{t}dt+\frac{1}{\sqrt{N}}ED(X_{t})dW+K_{t}.$$

Here **X**_**t**_ is a vector describing the proportion of channels in each state of the Markov formulation, **K**_**t**_ is a stochastic process that ensures each element of **X**_**t**_ remains in the interval 0 to 1 and **dW** is a vector of independent Wiener increments sampled from a Gaussian distribution with mean 0 and variance equal to the simulation time step. The first term represents the standard deterministic model of ion channel dynamics, where **A** is a matrix of the transition rates. The second term accounts for the stochastic fluctuations due to intrinsic noise and the magnitude of this term depends on the number of channels, **N**. The columns of the constant matrix **E** represent the changes to the number of channels in each state that occur as a result of the channel transitioning between states. **D**(**X**_**t**_) is a diagonal matrix whose entries correspond to the rate at which each transition occurs and so are a function of the vector **X**_**t**_. The form of the matrices **A**, **E** and **D**(**X**_**t**_) for each ion channel are given in the appendix (S1 Appendix: Formulation of the Ion Channel SDEs). Sample code of the numerical method used to simulate the dynamics of the reflected SDE is available for a neuronal cell model on the on the ModelDBwebsite (accession number 144489). This method represents an improvement with respect to the SDE approach taken in [17] since the reflected SDEs are formulated as a direct approximation of the underlying discrete-state channel dynamics model. The main advantage of our approach is that it preserves the distributional properties of the underlying discrete model [23], while this is not necessarily the case with the SDE approach presented in [17]. This could, at least partially, explain why the approach taken in [17] does not reproduce the shapes of the experimental Poincaré plots for control conditions.

#### Estimation of single channel current / unitary conductance

**Fig 1:**
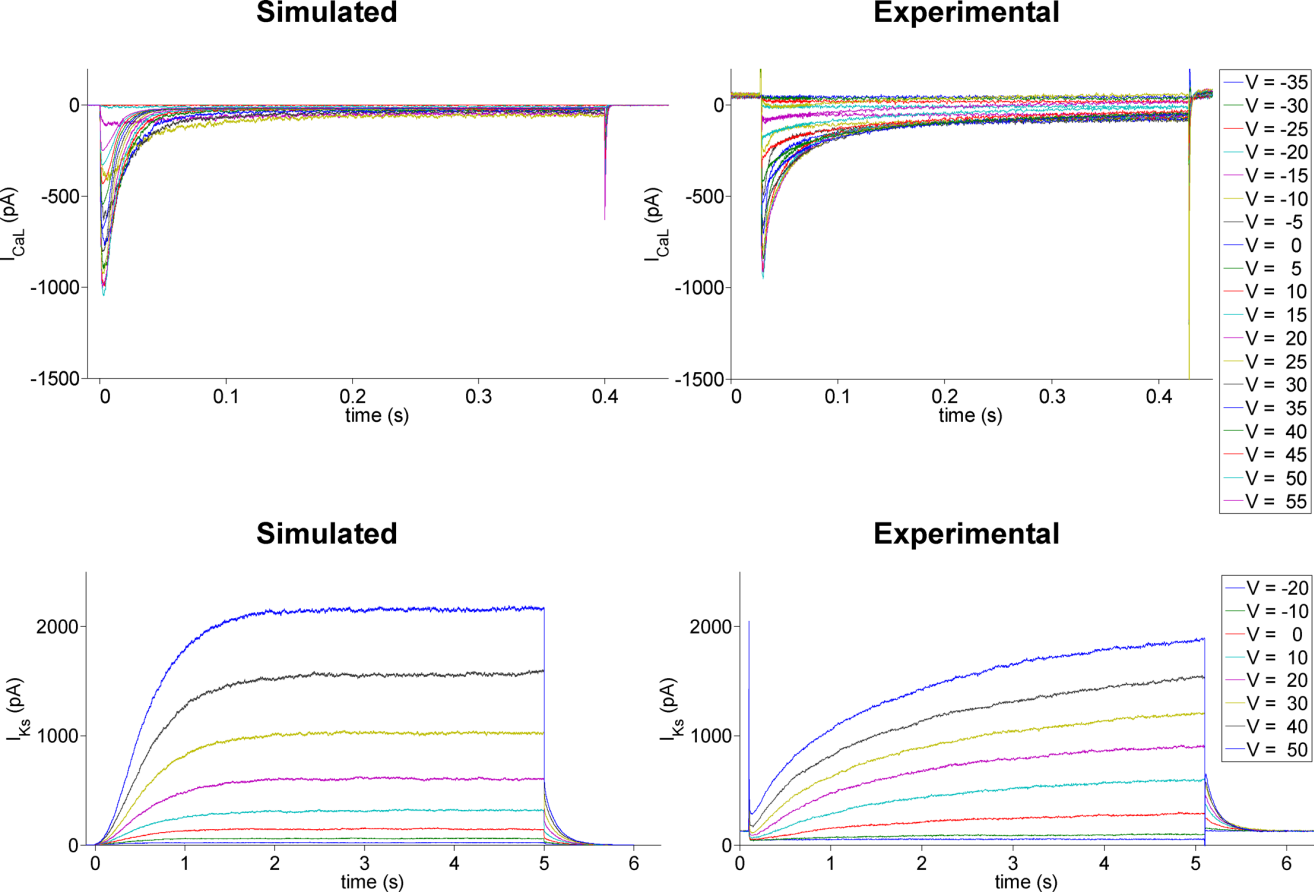
Simulated and experimental canine I_CaL_ and I_Ks_ traces. Examples of simulated and experimental canine I_CaL_ (top) and I_Ks_ (bottom) traces, following voltage pulses from -40 mV to test potentials ranging from -35 to 55 mV in 5-mV increments in the case of I_CaL_ and from -20 to 50 mV in 10-mV increments in the case of I_Ks_.

Ion channel numbers were computed for each deterministic model by obtaining estimates of the single channel current or unitary conductance from experimental recordings. For I_CaL_ and I_Ks_, single channel current values were estimated from voltage clamp experiments, as in [2]. For each test potential, seven experimental traces of each current were available. Stationary and nonstationary fluctuation analysis techniques were applied over those data and the single channel current for I_CaL_ and I_Ks_, denoted by i_CaL_ and i_Ks_, respectively, were calculated, obtaining estimated values of i_Ks_ = 0.15 pA at 40 mV and i_CaL_ = -0.6 pA at -15 mV. In Fig 1 examples of simulated and experimental ionic current traces of I_CaL_ and I_Ks_ are presented.

For I_to1_ and I_Kr_, a large enough number of clean current recordings could not be obtained from voltage clamp experiments due to the presence of residual currents or measurement noise, which rendered the traces unsuitable for the fluctuation analysis. Therefore unitary conductance values were obtained from the literature. To the best of our knowledge no such values have been reported for canine ventricular cells. For I_to1_, the unitary conductance value for rabbit ventricular myocytes reported in [24] was considered and correction for temperature was applied following the discussion in [24]. A value of 19.722 pS for I_to1_ unitary conductance was used to estimate N_to1_. For I_Kr_, a unitary conductance value of 2.5331 pS for human ventricular myocytes, obtained from [25] after conversion to an extracellular potassium concentration of 5.4 mM, was used to estimate N_Kr_. The conductance value is similar to values reported in other species: 2.4856 pS in rabbit ventricular myocytes [26], 3.4151 pS in mouse ventricular myocytes [27] and 2.8459 pS in guinea pig atrial myocytes [28], all of them obtained after conversion to an extracellular potassium concentration of 5.4 mM and, where necessary, corrected for temperature effects.

We note that the values obtained for the unitary conductances from the literature are very precise, as they are calculated as the slopes of linearly fitting experimental data representing single channel current versus voltage. It is however likely that such values lie within some physiological range due to extrinsic variability and experimental uncertainty. While, as in previous studies [16], we used the precise unitary conductance values reported in the literature, our approach, by considering a set of models, removes some of the limitations of using a single unitary conductance value to estimate the number of channels.

#### Estimation of channel numbers

From the estimates of single channel current for I_Ks_ and I_CaL_ (i_Ks_ and i_CaL_), the number of channels in each deterministic model of the calibrated set was calculated. For each model, I_Ks_ and I_CaL_ traces were simulated using the same voltage clamp protocol as in experiments. From the simulated macroscopic currents (I_Ks_ and I_CaL_) and channel open probabilities (p_Ks_ and p_CaL_) as well as the experimentally derived i_Ks_ and i_CaL_, the number of channels was calculated as: $N_{x}=\frac{I_{x}}{\left(i_{x}p_{x}\right)},$ for x = Ks, CaL, where N_x_ is the number of channels of type x, I_x_ is the current of type x, i_x_ is the single channel current of type x and p_x_ is the channel open probability of type x. On the other hand, N_to1_ and N_Kr_ were estimated by dividing the maximal conductances for each model in the set by the unitary conductance values obtained from the literature.

#### Stimulation protocols and data analysis

For each stochastic AP model, a train of 80 beats at steady-state was simulated at a BCL of 1000 ms, as in experiments, and repeated 50 times (a larger number of simulations altered the quantified measures only minimally). The last 30 beats of each train were used for the quantification of APD.

Four different measures of BVR reported in the literature were quantified in our study: APD range (ran_APD_), APD variance (var_APD_), short-term APD variability (STV_APD_) and long-term APD variability (LTV_APD_). We included STV_APD_ and LTV_APD_ in APD since they quantify variations between consecutive beats, which may be an important biomarker of arrhythmic risk [11]. The variability in APD was quantified over 30 beats and STV_APD_ was calculated as:

$${STV}_{APD}=\sum_{n=2}^{30}\frac{\left|{APD}^{n}-{APD}^{n-1}\right|}{\left(\sqrt{2}\times 29\right)},$$

where APD^n^ is the APD calculated at beat n. This is effectively the average distance perpendicular to the line of identity in the Poincaré plot, which is a plot of APD at beat n against beat n+1. LTV_APD_ was calculated as:

$${LTV}_{APD}=\sum_{n=2}^{30}\frac{\left|{APD}^{n}+{APD}^{n-1}-2\;\bar{APD}\right|}{\left(\sqrt{2}\times 29\right)},$$

where $\bar{\mathrm{APD}}$ is the mean APD value over the 30 beats. LTV_APD_ corresponds to the average distance along the line of identity in the Poincaré plot. The variable ran_APD_ was defined to be the difference between the maximum and minimum APD, while var_APD_ was calculated as the variance of APD values over the 30 beats. Linear partial correlation was used to identify correlations between the number of a specific type of ion channel and measures of BVR, while controlling for the effects of the numbers of other ionic channels, as in [3]. Such a method measures the strength of the linear relationship between two variables with the effect of one or more other variables removed.

## Results

### Construction of an experimentally-calibrated set of stochastic AP models

**Fig 2:**
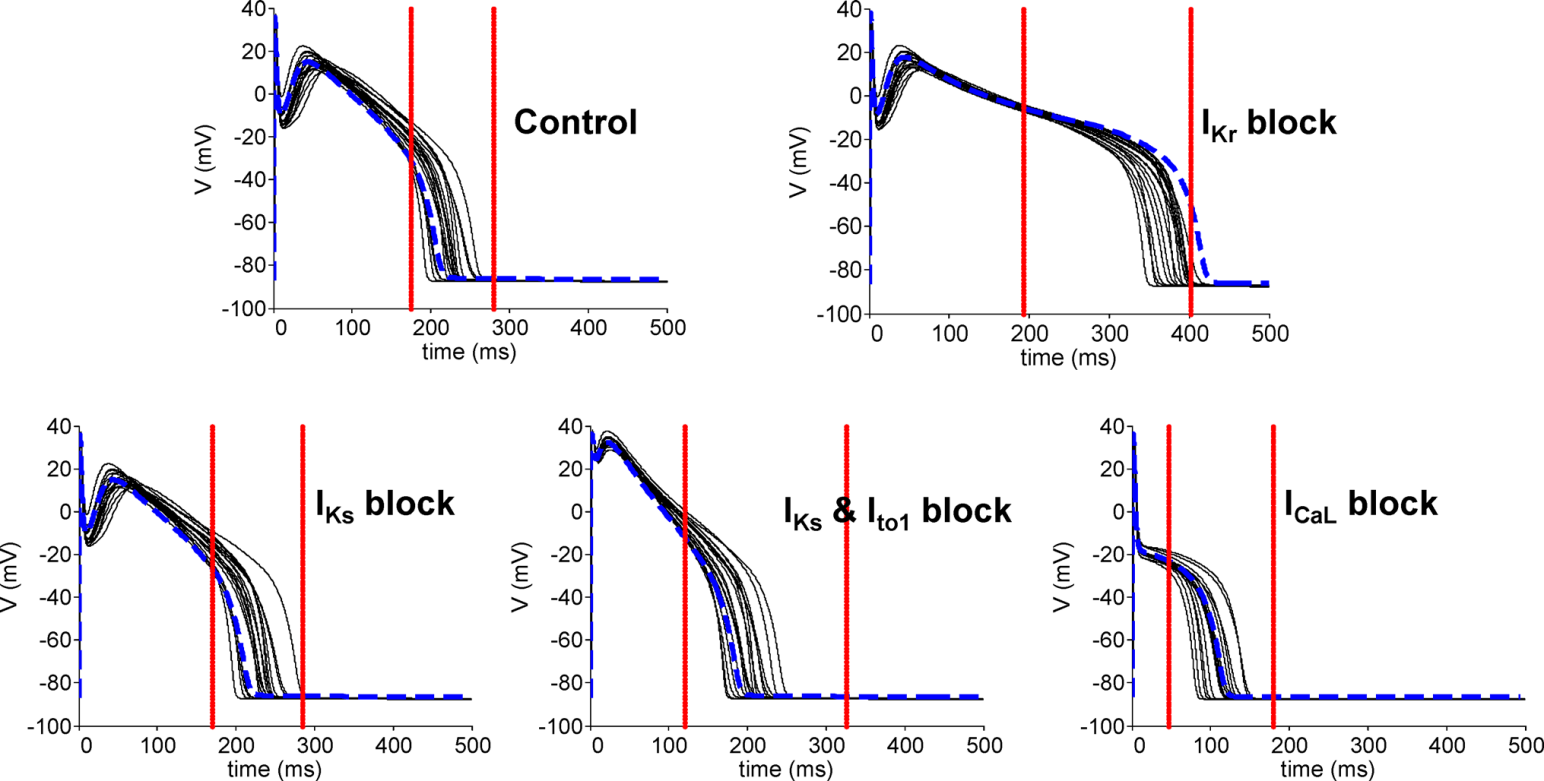
Deterministic model single AP traces. Single AP traces obtained using the seventeen deterministic models, simulated with a BCL of 1000 ms under: control conditions (top left), complete I_Kr_ inhibition (top right), complete I_Ks_ inhibition (bottom left), complete I_Ks_ and 90% I_to1_ inhibition (bottom middle) and 95% I_CaL_ inhibition (bottom right). Red vertical lines represent the maximum and minimum APD values derived from experimental data for the same simulation protocol. The blue dotted AP trace represents the Decker model without any rescaling of the ionic currents.

Following experimental calibration, 17 canine ventricular cell models based on the Decker model [21] were selected as being in range with APD experimental recordings in control and under the four ionic blocks. Fig 2 shows the final AP trace for each accepted model, with the red vertical lines indicating the maximum and minimum APD values derived from the experiments. Each panel illustrates results for one of the five different conditions used to calibrate the set of models, namely: control conditions (top left), complete I_Kr_ inhibition (top right), complete I_Ks_ inhibition (bottom left), complete I_Ks_ inhibition and 90% I_to1_ inhibition (bottom middle) and 95% I_CaL_ inhibition (bottom right). It is important to highlight that for the 17 models, the APD was in range (Fig 2) and also the change in APD produced by each of the four pharmacological interventions was in range with experimental data. The blue trace is the AP for the original Decker model, which results in a distinctly long AP in the presence of complete I_Kr_ inhibition in comparison with the experimental data used in this study. In this study, the set of models approach is primarily used as a methodology to capture differences in ionic conductances between a limited number of cells as in the experiments, without attempting to cover the whole experimental range. Since 80-beat traces were repeatedly simulated for each model in 50 stochastic simulations, 17 models also ensured computational tractability of the stochastic simulation study.

### Ranges in ion channel numbers and conductances across the set of models

**Fig 3:**
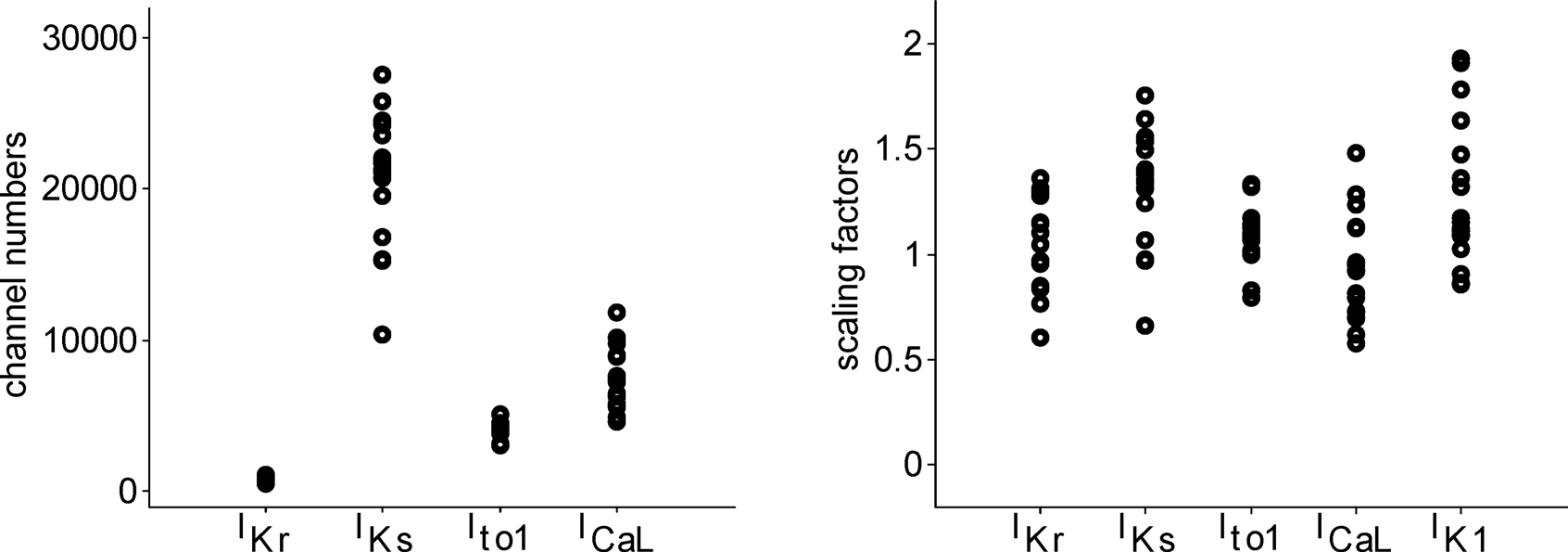
Channel numbers and current conductance scaling factors. Number of channels (left) and scaling factors for current conductances (right) in the seventeen models for I_Kr_, I_Ks_, I_to1,_ I_CaL_ and I_K1_.

Fig 3 shows the range in channel numbers across the set of models for each ionic current (left) and the scaling factors for each current conductance (right). The numbers of ion channels for each current will be referred to as N_Kr_, N_Ks_, N_to1_ and N_CaL_. As per the stochastic equation, they determine the magnitude of temporal variability in the proportion of open channels and thus the variability in the ionic current. Note that, for each ionic current, the current conductance in the model is directly proportional to the number of channels, as the single channel conductance is assumed to remain constant. Across the set of models N_Kr_ is consistently an order of magnitude smaller, in association with its very low maximal conductance in the Decker model, and the variability in channel numbers also smaller, as compared to the numbers of channels of the other currents. On the other hand, the variability in N_Ks_ and N_CaL_ is the greatest. The variability in scaling factors (right panel) is similar for all the currents, with the variability in I_Ks_ scaling being the greatest.

### Stochastic models reproduce experimental BVR measures

**Fig 4:**
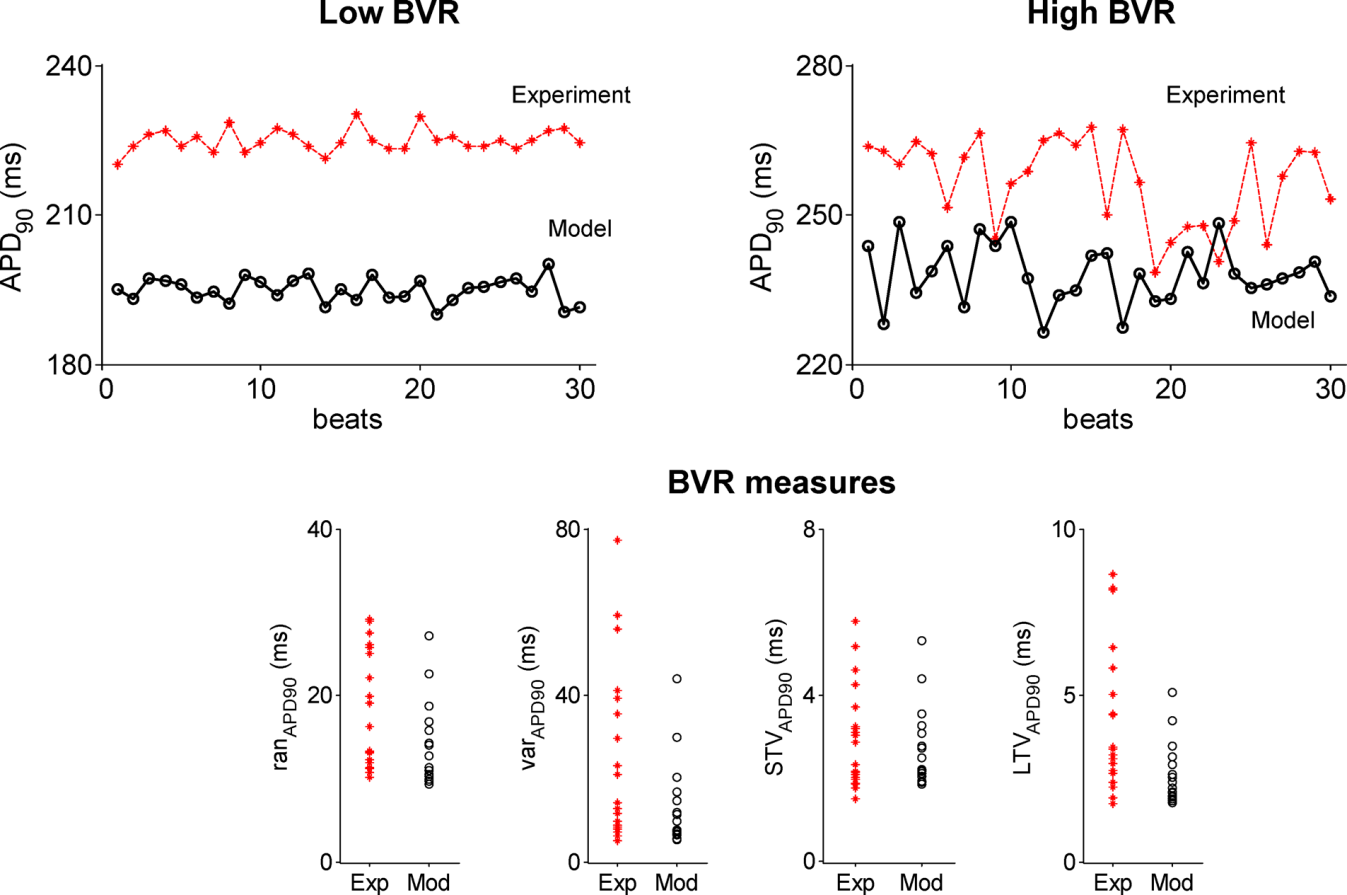
Results from population of stochastic models under control conditions. Top: APD sequences of 30 beats for individual experiments and model realizations under control, illustrating cases associated with low (left) and high (right) temporal variability. Bottom: BVR measures in experiments (red crosses) and simulations (black open circles), calculated in mean over realizations, under control conditions. The BVR measures, from left to right, are as follows: ran_APD_, var_APD_, STV_APD_ and LTV_APD_.

We then conducted stochastic simulations to investigate the role of stochasticity in I_CaL_, I_Kr_, I_Ks_ and I_to1_ on the four measures of BVR under control conditions. The top panels of Fig 4 show experimental APD sequences with low (left) and high (right) BVR and how this is reproduced by individual stochastic models. Both the experimental and simulated examples shown in Fig 4 were selected as correspondent to the lower and upper quartiles of all analysed cases. Also shown in Fig 4 (bottom panels) are the four variability measures for each experiment and each model in the set (in mean over realizations). Importantly, simulated BVR measures are in range with experimental BVR. In particular, for the measures ran_APD_ and STV_APD_ simulated values represent the entire experimental range. For var_APD_ and LTV_APD_, the simulations cover the range where the majority of experimentally recorded values lie, however the largest values are not captured in the simulations. Additionally, the simulated STV_APD_-to-LTV_APD_ ratios, describing the shape of the Poincaré plot, are also in agreement with experimental values.

**Fig 5:**
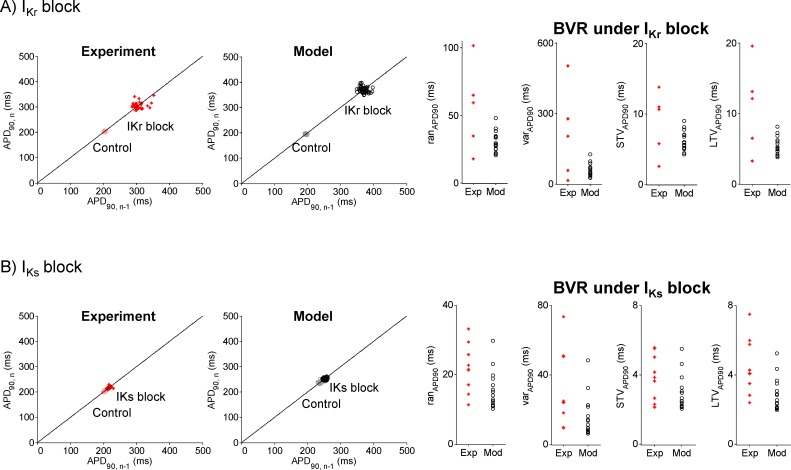
Results from population of stochastic models under I_Kr_ and I_Ks_ inhibition. A) Left: Poincaré plots for an individual experiment and a model realization under control and following I_Kr_ inhibition. Right: BVR measures in experiments (red crosses) and simulations (black open circles), calculated in mean over realizations, under I_Kr_ inhibition. The BVR measures, from left to right, are as follows: ran_APD_, var_APD_, STV_APD_ and LTV_APD_. B) As in A) but following I_Ks_ inhibition.

Furthermore, all the BVR measures as a result of complete I_Kr_ inhibition or complete I_Ks_ inhibition are also in accordance with experimental recordings (Fig 5A and 5B, right panels). This also applies to the changes due to either of the two blocks and to the STV_APD_-to-LTV_APD_ ratio, except for the case of I_Kr_ inhibition where the simulated ratios are somewhat larger than in the experiments. Therefore Fig 4 and Fig 5 confirm that the set of stochastic models reproduces experimentally-observed levels of beat-to-beat variability in APD under both physiological and pharmacological conditions. Furthermore, as the two measures obtained from the Poincaré plots, namely STV_APD_ and LTV_APD_, are in accordance with experimental values, this suggests that the simulated Poincaré plots are also similar to those obtained from experiments. This is illustrated in the left panels of Fig 5A and 5B, showing representative examples of experimental and simulated Poincaré plots for control and following I_Kr_ and I_Ks_ inhibition, respectively. The agreement between experiments and simulations confers credibility to the set of stochastic models calibrated and evaluated with different experimental measurements.

### Stochasticity in I_to1_ is the largest contributor to BVR

**Fig 6:**
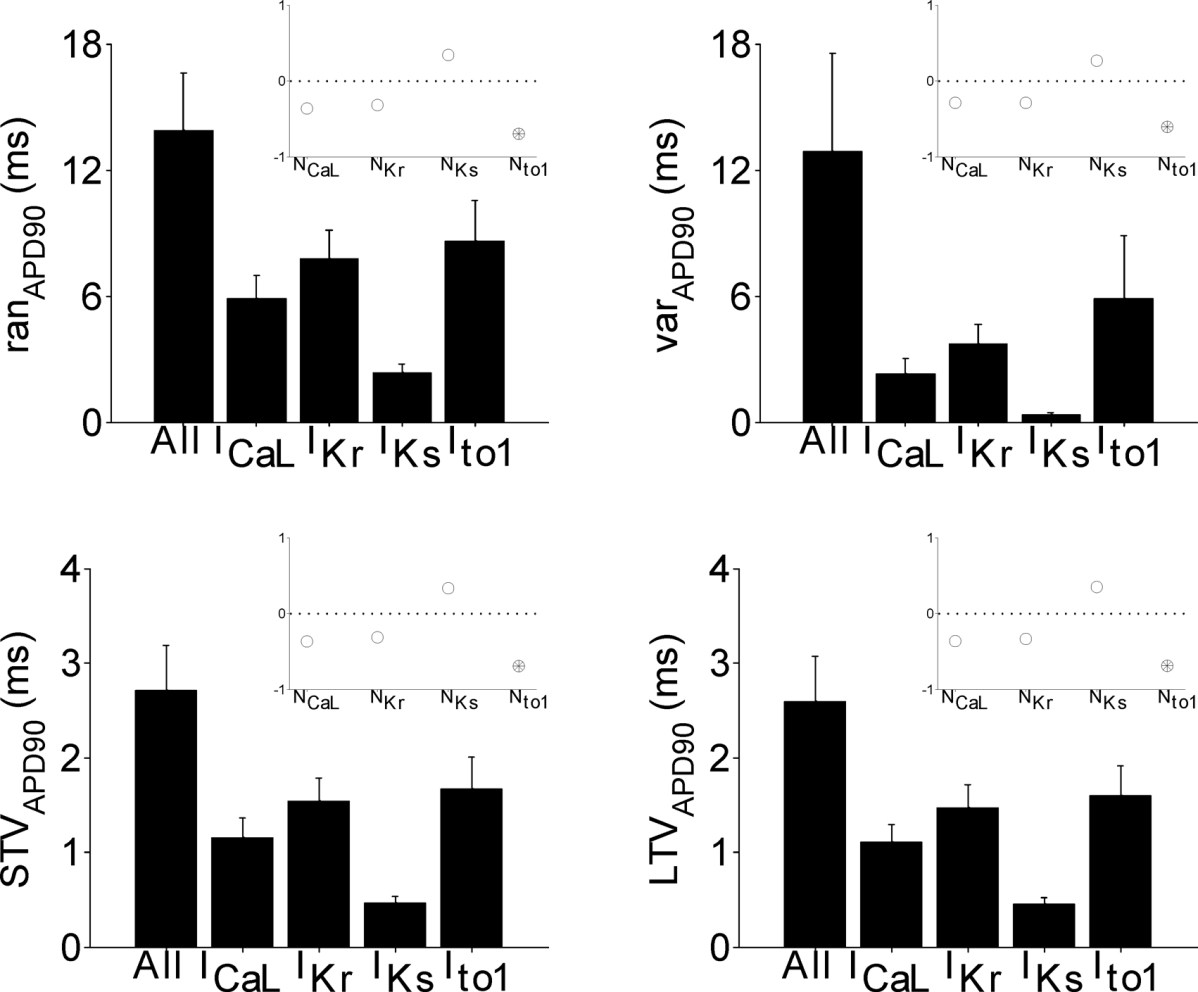
Variability in APD across the population of models under control conditions. Bar graphs showing the mean and standard deviation of APD variability measurements across the seventeen models from simulations where all currents are stochastic and also when stochasticity is incorporated into each individual current, under control conditions. The measures of variability are ran_APD_ (top left), var_APD_ (top right), STV_APD_ (bottom left) and LTV_APD_ (bottom right). Insets: Partial correlation coefficients between variability measures and ion channel numbers, with black dots and open circles indicating significant and not significant correlation, respectively.

Fig 6 describes the contribution of stochasticity in each ionic current individually to BVR. Bar graphs show the mean and standard deviation for each BVR measure over the entire set of models, with stochasticity in all currents compared to models with stochasticity introduced in each individual current. The stochastic behaviour of I_to1_ contributes the most to all measures of BVR, with the stochastic behaviour of I_to1_ alone able to reproduce 62% of ran_APD_, 46% of var_APD_, 62% of STV_APD_ and 62% of LTV_APD_. I_Kr_ is the next largest contributor to all measures of BVR, while I_CaL_ and I_Ks_ contribute the least. Furthermore, in some cases each current alone can reproduce more than 1/4 of the variability observed when all currents are stochastic, and so their contribution cannot be described as a simple additive relationship.

### N_to1_ is an important determinant of BVR under control conditions

Fig 6 also shows the results of the partial correlation analysis between the measures of BVR and ion channel numbers in control (insets). Significant partial correlation coefficient values are shown as black dots, while open circles mean that the partial correlation was deemed insignificant (that is, it had a p-value greater than 0.05). As shown in Fig 6, there is a strong negative correlation between N_to1_ and all four measures of BVR. N_CaL_ and N_Kr_ are non-significantly (negatively) correlated with the BVR measures, meaning that they do not independently influence BVR substantially. N_Ks_ is non-significantly (positively) correlated with BVR, also indicating a weak independent influence on BVR, as in the case of N_CaL_ and N_Kr_.

### Stochasticity in I_Ks_ and I_CaL_ are the largest contributors to BVR following I_Kr_ inhibition

**Fig 7:**
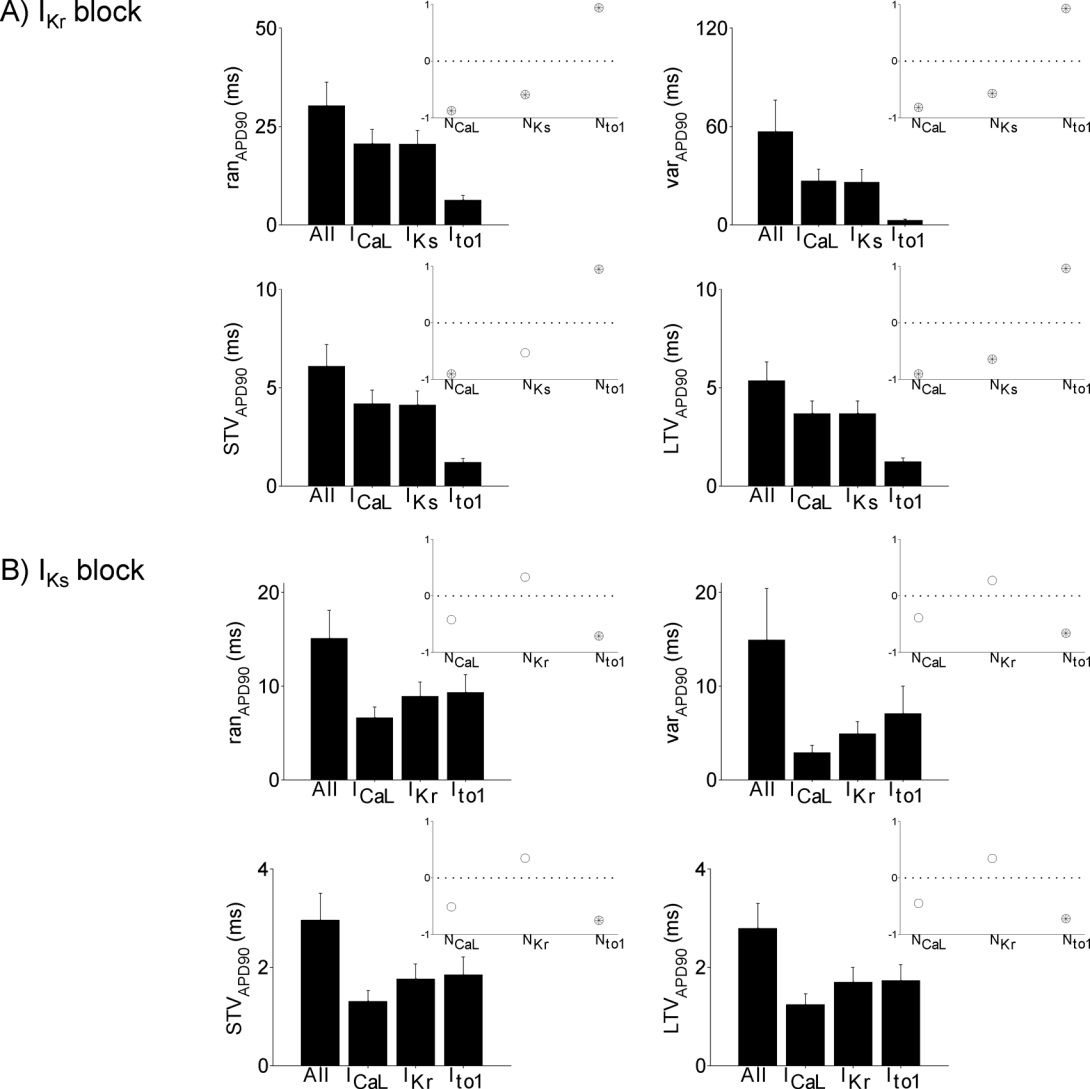
Variability in APD across the population of models under I_Kr_ and I_Ks_ inhibition. A) Bar graphs showing the mean and standard deviation of APD variability measurements across the set of models from simulations where all currents are stochastic and also when stochasticity is incorporated into each individual current, following I_Kr_ inhibition. The measures of variability are ran_APD_ (top left), var_APD_ (top right), STV_APD_ (bottom left) and LTV_APD_ (bottom right). Insets: Partial correlation coefficients between variability measures and ion channel numbers, with black dots and open circles indicating significant and not significant correlation, respectively. B) As in A) but following I_Ks_ inhibition.

Following complete I_Kr_ inhibition, simulation results show that stochasticity in I_Ks_ and I_CaL_ are the largest contributors to BVR (bar graphs in Fig 7A) with both of similar relevance. On the other hand, the inhibition-induced change in BVR is largely driven by the stochasticity in I_Ks_. In fact, upon complete inhibition of I_Kr_, the change in BVR measured when I_Ks_ is the only stochastic current is larger than when all currents are stochastic for three of the BVR measures (ran_APD_, STV_APD_ and LTV_APD_, not shown). Stochasticity in I_CaL_ is the second largest contributor. Regarding complete inhibition of I_Ks,_ no significant differences between the contributions of stochasticity in the currents before and after inhibition were found (Fig 7B).

### N_to1_, N_CaL_ and N_Ks_ are major determinants of BVR following I_Kr_ inhibition

Following complete inhibition of I_Kr_, N_to1_ and BVR show strong positive correlation for all BVR measures (Fig 7A). This is in contrast to the negative correlation present under control conditions (Fig 6). Therefore, under control conditions, smaller N_to1_ leads to greater BVR, but upon inhibition of I_Kr_, larger N_to1_ results in greater BVR. These results suggest that under control conditions the increased magnitude of I_to1_ fluctuations caused by smaller channel numbers is more important in BVR than the individual contribution of this current to the AP, while this is reversed upon inhibition of I_Kr_.

Under control conditions there was no significant relationship between N_CaL_ or N_Ks_ and any BVR measures (Fig 6). However, after complete I_Kr_ inhibition we found a strong negative correlation between N_CaL_ or N_Ks_ and practically all measures of BVR (Fig 7A, insets) as well as the change in BVR. This suggests that under physiological conditions N_CaL_ or N_Ks_ do not independently influence BVR, but under conditions of reduced repolarization reserve, such as I_Kr_ inhibition, N_CaL_ and N_Ks_ become important determinants of BVR.

### N_to1_ exerts the strongest influence on BVR following I_Ks_ inhibition

Upon complete I_Ks_ inhibition, we found similar relationships between the channel numbers of each of the remaining currents and BVR as reported for control conditions. The only parameter found to significantly correlate with BVR measures and their change after I_Ks_ inhibition was N_to1_, for which there was a strong negative correlation (Fig 7B, insets). The strength of the relationships between the number of channels of each ionic current (N_CaL_, N_Kr_, N_to1_) and BVR or the change in BVR after I_Ks_ inhibition was similar to that in control. Altogether, these results suggest that temporal variability and its change as a result of I_Ks_ inhibition is primarily driven by N_to1_.

## Discussion

In this study, we develop a methodology to construct experimentally-calibrated sets of stochastic canine AP models and we investigate the contribution of four main ionic currents (I_Kr_, I_Ks_, I_to1_, I_CaL_) to several measures of BVR in control and following pharmacological ionic channel inhibition. Experimental AP and voltage clamp recordings are used to construct and calibrate a set of stochastic ventricular AP models that considers cell-to-cell differences in ion channel densities. Simulations using the experimentally-calibrated stochastic models, based on reflected SDEs, are evaluated using additional data for control and following pharmacological action (not used for calibration), and further studies are conducted to identify key ionic mechanisms underlying BVR.

In addition to the methodological novelties of our study, the main physiological findings of our simulation study are:

1. under physiological conditions and after complete I_Ks_ inhibition, I_to1_ is the largest contributor to BVR out of the 4 analyzed currents, with I_Kr_ being the second largest contributor;
2. the contributions of individual currents to BVR combine in a non-additive way;
3. under complete I_Kr_ inhibition, I_Ks_ and I_CaL_ become major contributors to BVR and are also important in the increase in BVR;
4. N_to1_ is strongly negatively correlated with BVR, while N_CaL_, N_Kr_ and N_Ks_ do not independently affect BVR under physiological conditions;
5. after complete I_Kr_ inhibition, the relationship between N_to1_ and BVR is reversed, with N_to1_ becoming strongly positively correlated with BVR;
6. upon I_Kr_ inhibition, N_to1_, N_CaL_ and N_Ks_ are all independent determinants of BVR and of the increase in BVR;
7. after I_Ks_ inhibition, only N_to1_ seems to independently affect BVR and its increase with respect to control.

Our simulations using experimentally-calibrated models show that fluctuations in the four considered currents reproduce a large part of the BVR measured in canine ventricular myocytes. This is consistent with the known importance of the four currents in the repolarization phase of canine ventricular cardiomyocytes. Additional mechanisms such as stochasticity in the sodium current or calcium dynamics, as identified in previous studies [17], may also contribute to BVR. The methodology presented in this work can be extended to include additional mechanisms in further studies as long as the required experimental data are available for corresponding model development and validation, therefore including ionic current measurements and action potential recordings with selective pharmacological block.

Our simulations demonstrate that the experimentally-calibrated stochastic models yield BVR values consistent with experiments for four different measures: ran_APD_, var_APD_, STV_APD_ and LTV_APD_. In contrast to the results found by Heijman et al. when using SDEs to model stochastic I_Kr_ gating, who reported simulated Poincaré plot shapes markedly different from the circular shapes recorded experimentally under physiological conditions [17], in the present study the STV_APD_-to-LTV_APD_ ratio is in accordance with experimental ranges, confirming that agreement exists between simulated and experimental shapes of the Poincaré plots under such conditions. While Heijman et al. were able to reproduce experimentally recorded STV_APD_-to-LTV_APD_ ratio using stochastic gating, such an approach is much more computationally intensive than the SDE approach described in the present study. Furthermore, simulation time for our SDE approach does not increase with the number of channels or the number of states [23], and so can be more easily extended to complex models than the stochastic gating approach used in [17]. This is an important methodological novelty. Differences in the construction of the sets of models may underlie our improvements. Indeed Heijman et al. introduced I_Kr_ stochasticity in the otherwise deterministic Decker model, while we considered a set of experimentally-calibrated deterministic cell models (each associated with a different combination of ionic conductances and ion channel numbers). In our study, the baseline Decker model (without altering ionic conductances) did not reproduce our experimental recordings and therefore did not satisfy the calibration criteria. Also, differences between the two studies can be found in the methods used to formulate and solve the SDEs. Here we introduced reflected SDEs to model ion channel dynamics, which ensure preservation of the stochastic dynamics of discrete-state Markov chain models while providing biologically realistic solutions [23].

We have found that, under physiological conditions, I_to1_ is the major contributor to all the investigated BVR measures, with I_Kr_ being the second largest contributor. This is in partial agreement with the results reported by Heijman et al., who also incorporated stochastic effects in the sodium current and identified a major role of the persistent sodium component, together with I_Kr_, in determining BVR in canine ventricle cardiomyocytes [17]. The role of I_to1_ in [17] was less relevant than in the present study, and we believe this may be due to differences in the methodologies and assumptions in the models. One important methodological difference is that Heijman et al. assessed the contribution of stochastic gating and current conductances in different sets of simulations and assumed that a change in ion channel numbers was associated with a reciprocal change in single channel conductances. On the contrary, we investigated the main contributors to BVR by assuming that single channel conductances are constant (and remain unchanged), whereas ionic current conductances and channel numbers vary in a proportional manner, as they are necessarily related, which is in agreement with experimental findings [29]. Also, the number of I_to1_ channels in [17] was 5900, while in our study the number of I_to1_ channels in the calibrated population ranged from approximately 3000 to 5000. In the calculation of channel numbers we used experimentally reported I_to1_ unitary conductance values after conversion to physiological temperature rather than considering the values corresponding to room temperature, as in [17].

With our approach we were also able to recognize the contribution of currents for which both stochastic channel gating and conductance variations are relevant to BVR. To further assess BVR determinants, we computed partial correlation coefficients between BVR and ion channel numbers. We found that BVR was strongly negatively correlated with N_to1_ under physiological conditions. While smaller channel numbers result in greater temporal fluctuations of the ionic current, the contribution of this current to the overall membrane potential will also be less. Furthermore, the contribution of each current to the membrane potential affects the APD and previous studies have shown that APD is a key determinant in the magnitude of BVR [8], [17]. We conducted additional simulations in which N_to1_ was varied in the same range of the initial simulations but the maximal I_to1_ conductance was kept constant and we confirmed (not shown) that the importance of N_to1_ in determining BVR was in large part due to fluctuations in the I_to1_ current and to a lesser extent due to I_to1_ conductance modulation of the AP.

The second largest contributor to BVR under physiological conditions was I_Kr_. As mentioned above, this agrees with the results reported in [17]. Altomare et al. also described the impact of I_Kr_ on BVR and found that the major effect was related to I_Kr_ inactivation [30]. Lemay et al. did not find a notable role of I_Kr_ in BVR, but this can be explained by inter-species differences, as I_Kr_ (and the associated number of channels) is much larger in the guinea pig Faber-Rudy model used in [16] than in the canine Decker model used in the present study. Despite the contribution of I_Kr_ stochasticity to temporal variability, when analysing the partial correlation between BVR and N_Kr_, we did not find significance in such a relationship. This may be explained either because N_Kr_ and BVR measures are not linearly correlated or most likely because the correlation effects are already accounted for by the contribution of other ionic currents. In fact, in our simulations, BVR measures with stochasticity in all our analysed currents except for I_Kr_ are around 20% smaller than variability levels measured when stochasticity is included in all currents. Our results confirm that variability introduced by I_Kr_ stochasticity is to a good extent accounted for by the contribution of all the other currents.

With regard to I_CaL_ and I_Ks_, we found that their contribution to BVR was considerably lower than that of I_to1_ and I_Kr_. In canine cardiomyocytes, Heijman et al. reported a minor effect of I_CaL_ on temporal variability as well, with an even less relevant influence than in the present study, which can be related to the larger number of I_CaL_ channels used in [17] as well as to the mutual effects of other ionic currents. In particular, the number of I_CaL_ channels in our calibrated population ranged from approximately 4500 to 12000 channels, based on our single-channel current estimates from canine ventricular myocytes, while in [17] a much larger number of I_CaL_ channels was calculated using experimentally reported unitary conductances in rat ventricle [31]. Our population of canine ventricular models would render single-channel current values lying between 0.2 and 0.6 pA at 0 mV, while Guia et al. reported values around 0.12 pA in rat ventricular myocytes [31]. As regards I_Ks_ stochasticity, despite having some contribution to temporal variability measures, in agreement with the results of previous studies in guinea pig and human [16], [2], we did not find significance in the partial correlation between BVR measures and the number of I_Ks_ channels, probably as it is concealed by other ionic currents playing a more relevant role in the canine ventricle. Consistent with these results, Heijman et al. also found some contribution of I_Ks_ stochasticity to BVR, although with a less predominant role than other currents [17]. The number of I_Ks_ channels in [17] was 10500 channels, calculated from unitary conductance values reported in heterologous expression systems, while we estimated single-channel current values in canine ventricular myocytes, still within the range reported in recent studies on heterologous expression systems [32], which led to a range of channel numbers in our calibrated model population varying from around 10000 to 27000 channels.

As well as investigating cell-to-cell differences in BVR under physiological conditions we also studied the effects of I_Kr_ or I_Ks_ inhibition on BVR. Previous computational studies have shown that a reduction in I_Kr_ leads to an increase in simulated BVR [2], [16]. Here we are able to extend these findings, as the set of stochastic models allows for investigation into the ionic basis for such an increase. In particular, we found that this was primarily driven by I_Ks_ and I_CaL_ rather than I_to1_. In terms of partial correlation between BVR measures and ion channel numbers, smaller N_Ks_ and N_CaL_ led to greater variability under I_Kr_ inhibition, while N_to1_ was positively correlated with BVR. The correlation results for N_to1_ are in sharp contrast to those found in control conditions, where N_to1_ showed a strong negative correlation with BVR. Results (not shown) of additional simulations where the maximal I_to1_ conductance was kept constant corroborated that upon blockade of I_Kr_ the main driver of BVR and of its increase with respect to control is I_to1_ conductance modulation of the AP rather than I_to1_ current fluctuations. We additionally investigated the ionic basis for the increase in BVR as a result of I_Ks_ inhibition and found that N_to1_ was the major independent determinant for this increase in BVR, closely resembling the results found in control. Since I_Ks_ block only slightly altered the AP in control, the similarity in terms of ionic contributors to BVR between control and I_Ks_ block conditions could be to a large extent expected and was corroborated in the present study.

The BVR simulated with the experimentally-calibrated set of models reproduces a large part of the experimental range for all measures of variability, both in control and under complete I_Kr_ and I_Ks_ inhibition. The limited number of models considered, due to the computational constraints of stochastic simulations, limits the strength of the statistical analysis, which is why the values of correlation coefficients are presented even for those values found not to be statistically significant. However, previous modelling studies have similarly used a limited number of models (19) to investigate variability [33], and this is also comparable to the number of experimental samples used.

This study could be extended in a number of ways. Our study focuses on the contribution of four ionic currents to BVR. Additional sources of stochasticity could be implemented and investigated using our approach in further studies, provided the experimental measurements for model calibration and evaluation are available. This would be particularly important for currents, such as the persistent sodium current, which might contribute to the BVR_,_ particularly under pharmacological conditions [34]. We focused on assessing the effects of I_Kr_, I_Ks_, I_to1_, I_CaL_ because they have a large impact on repolarisation and we had a consistent experimental dataset including ionic current measurements, and AP recordings under control and pharmacological block of those currents. This allowed us to construct and calibrate a whole population of models rather than just considering a unique AP model, like the Decker model. The Decker model was, in fact, not included in our population because it did not lead to APD values in range with our experimental recordings in control as well as following ionic inhibitions. The model population we developed allows investigating the relative importance of each of the analysed currents in contributing to BVR taking into consideration variability in ionic conductances and channel numbers. In the case of the persistent sodium current (I_NaL_) we did not have available current traces and AP data measured under selective I_NaL_ inhibition that we could use to construct and calibrate the models following the same procedure that we used with all the other analysed currents. Future studies could assess the role of stochasticity in I_NaL_ in generating BVR using our proposed methodology as long as all necessary data are available, building on the methodology described in our study. Our experimentally-calibrated population of models was able to reproduce experimental observations regarding changes on BVR following sodium channel inhibition and enhancement [35, 36, 37, 38, 39], although widely varied responses could be quantified as a function of the analysed model (cell), degree of inhibition / enhancement as well as selectivity for both I_NaL_ and fast sodium current (I_Na_) or for I_NaL_ only.

Furthermore, the focus of the study has been at the cellular level as BVR is enhanced without gap junctional coupling and therefore it conveys important information as a biomarker of repolarization reserve. For investigations into the pro-arrhythmic causal relationship of BVR, tissue simulations built on the models developed in this study could be considered.

## Supporting Information

S1 Appendix Formulation of ion channel SDEs. Detailed description of the method used to formulate the stochastic differential equations used to describe ion channel dynamics. The full formulas for the coefficients of these stochastic differential equations are also provided.
(PDF)

S2 Appendix Experimentally-based calibration. Experimental ranges used in the construction of the population of models: a) For normalized maximal IKr, IKs, Ito1 and ICaL current values. b) For APD and change in APD values under control conditions and following pharmacological inhibitions (complete IKr inhibition, complete IKs inhibition, 95% inhibition of ICaL and complete inhibition of IKs combined with 90% inhibition of Ito1).
(M) Click here for additional data file.

## Abbreviations

BVR: Beat-to-beat variability in repolarization duration
APD: Action potential duration
APD_90_: action potential duration at 90% of repolarization
SDE: Stochastic differential equation
ran_APD_: Range in action potential duration
var_APD_: Action potential duration variance
STV_APD_: Short-term APD variability
LTV_APD_: Long-term APD variability
