# Correction: Experimentally-Based Computational Investigation into Beat-To-Beat Variability in Ventricular Repolarization and Its Response to Ionic Current Inhibition

**DOI:** 10.1371/journal.pone.0197871

**Published:** 2018-05-17

**Authors:** E. Pueyo, C. E. Dangerfield, O. J. Britton, L. Virág, K. Kistamás, N. Szentandrássy, N. Jost, A. Varró, P. P. Nánási, K. Burrage, B. Rodríguez

The following information is missing from the Funding section: E.P. acknowledges the financial support of the European Research Council (ERC) through project ERC-2014-StG 638284.
