# Supplementary material for: Experimentally-Based Computational Investigation into Beat-To-Beat Variability in Ventricular Repolarization and Its Response to Ionic Current Inhibition
Source: PLoS One. 2016 Mar 28;11(3):e0151461. doi: 10.1371/journal.pone.0151461 (PMC4809506; doi:10.1371/journal.pone.0151461)
Supplement: S1 Appendix — Detailed description of the method used to formulate the stochastic differential equations used to describe ion channel dynamics. The full formulas for the coefficients of these stochastic differential equations are also provided. (PDF) [file pone.0151461.s001.pdf]

S1 Appendix: Formulation of Ion Channel SDEs

The equations in the Decker model that describe the dynamics of the following repolarisation currents I_{Ks}, I_{CaL}, I_{Kr} and I_{To1} were altered to include descriptions of the intrinsic stochastic behaviour of these channels. This same modification was made to each of the models within the population of deterministic models.

The Decker model describes the kinetics of I_{Ks} and I_{CaL} using a Markov formulation according to the state diagrams given in Figures 1 and 2, respectively. I_{Ks} is assumed to possess two distinct open states (O_1 and O_2), as is I_{CaL} (O and $O^{\star}$). The kinetics of the remaining time dependent ionic currents are described using the classical Hodgkin-Huxley formulation.

Since I_{Ks} and I_{CaL} are described using a Markov formulation, the stochastic model for these channels was constructed based on their respective kinetic diagrams, given in Figures 1 and 2, in terms of reflected SDEs. The states O_1, O_2) and O, $O^{\star}$ for the I_{Ks} and I_{CaL} channels respectively are assumed to be the only states in which the channels can conduct ions. All other states represent various different closed or inactive forms of the channel, such that the channel does not conduct ions in these states.

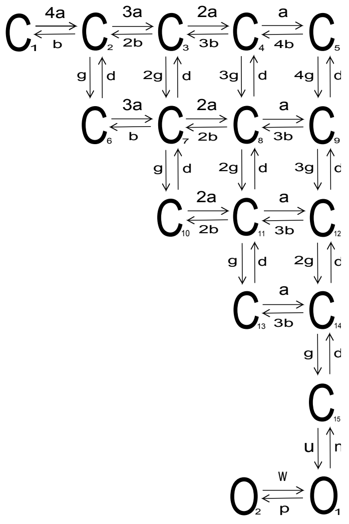

Figure 1: State diagram of the I_{Ks} dynamics in the Decker model.

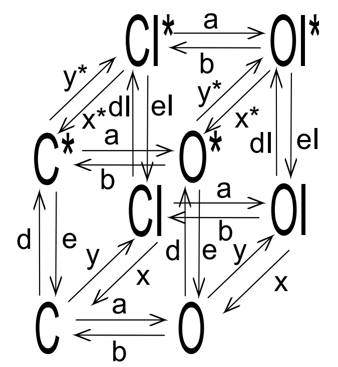

Figure 2: State diagram of the I_{CaL} dynamics in the Decker model.

The reflected SDEs describing the stochastic dynamics of I_{CaL} and I_{Ks} therefore take the following form,

$$d\boldsymbol{y}_{CaL} = H_{CaL}\boldsymbol{y}_{CaL}dt + \frac{1}{\sqrt{N_{CaL}}}E_{CaL}F_{CaL}(\boldsymbol{y}_{CaL})d\boldsymbol{W}_{CaL} + d\boldsymbol{K}_{CaL}, \tag{1}$$

$$d\boldsymbol{y}_{Ks} = H_{Ks}\boldsymbol{y}_{Ks}dt + \frac{1}{\sqrt{N_{Ks}}}E_{Ks}F_{Ks}(\boldsymbol{y}_{Ks})d\boldsymbol{W}_{Ks} + d\boldsymbol{K}_{Ks}, \tag{2}$$

where N_x is the number of channels of ion type x, $x = CaL$, Ks, and the matrices H_x, F_x, E_x, $x = CaL$, Ks, are given at the end of this section.

On the other hand, I_{Kr} and I_{To1} are modelled using the Hodgkin-Huxley formulation, where I_{Kr} is assumed to posses a single activation gate X_r and I_{To1} is described by three identical activation gates (a), a fast inactivation gate (i_f) and a slow inactivation gate (i_s). Incorporating stochasticity directly into the Hodgkin-Huxley formulation of ion channel dynamics can lead to inaccuracies in the stochastic dynamics [1, 2]. Therefore I_{Kr} and I_{To1} kinetics were reformulated in terms of the equivalent Markov formulations by assuming that each combination of gating positions is a distinct channel state, and so the dynamics of I_{Kr} and I_{To1} are described by the following kinetic diagrams respectively:

$$X_{r,0} \underset{\beta_{X_r}}{\overset{\alpha_{X_r}}{\rightleftarrows}} X_{r,1}$$

$$\begin{array}{ccccccc}
a_0 i_{f,0} i_{s,1} & & a_1 i_{f,0} i_{s,1} & & a_2 i_{f,0} i_{s,1} & & a_3 i_{f,0} i_{s,1} \\
\alpha_s \uparrow\downarrow \beta_s & & \alpha_s \uparrow\downarrow \beta_s & & \alpha_s \uparrow\downarrow \beta_s & & \alpha_s \uparrow\downarrow \beta_s \\
a_0 i_{f,0} i_{s,0} & \underset{\beta_a}{\overset{3\alpha_a}{\rightleftarrows}} & a_1 i_{f,0} i_{s,0} & \underset{2\beta_a}{\overset{2\alpha_a}{\rightleftarrows}} & a_2 i_{f,0} i_{s,0} & \underset{3\beta_a}{\overset{\alpha_a}{\rightleftarrows}} & a_3 i_{f,0} i_{s,0} \\
\beta_f \uparrow\downarrow \alpha_f & & \beta_f \uparrow\downarrow \alpha_f & & \beta_f \uparrow\downarrow \alpha_f & & \beta_f \uparrow\downarrow \alpha_f \\
a_0 i_{f,1} i_{s,0} & \underset{\beta_a}{\overset{3\alpha_a}{\rightleftarrows}} & a_1 i_{f,1} i_{s,0} & \underset{2\beta_a}{\overset{2\alpha_a}{\rightleftarrows}} & a_2 i_{f,1} i_{s,0} & \underset{3\beta_a}{\overset{\alpha_a}{\rightleftarrows}} & a_3 i_{f,1} i_{s,0} \\
\beta_s \uparrow\downarrow \alpha_s & & \beta_s \uparrow\downarrow \alpha_s & & \beta_s \uparrow\downarrow \alpha_s & & \beta_s \uparrow\downarrow \alpha_s \\
a_0 i_{f,1} i_{s,1} & \underset{\beta_m}{\overset{3\alpha_m}{\rightleftarrows}} & a_1 i_{f,1} i_{s,1} & \underset{2\beta_m}{\overset{2\alpha_m}{\rightleftarrows}} & a_2 i_{f,1} i_{s,1} & \underset{3\beta_m}{\overset{\alpha_m}{\rightleftarrows}} & a_3 i_{f,1} i_{s,1} \\
\alpha_f \uparrow\downarrow \beta_f & & \alpha_f \uparrow\downarrow \beta_f & & \alpha_f \uparrow\downarrow \beta_f & & \alpha_f \uparrow\downarrow \beta_f \\
a_0 i_{f,0} i_{s,1} & \underset{\beta_m}{\overset{3\alpha_m}{\rightleftarrows}} & a_1 i_{f,0} i_{s,1} & \underset{2\beta_m}{\overset{2\alpha_m}{\rightleftarrows}} & a_2 i_{f,0} i_{s,1} & \underset{3\beta_m}{\overset{\alpha_m}{\rightleftarrows}} & a_3 i_{f,0} i_{s,1}
\end{array}.$$

where α_y and β_y are the closed to open and open to closed transition rates, respectively, for gate of type y and y_i denotes the state where i gates of type y are open. The stochastic dynamics of the ion channels for I_{Kr} and I_{To1} are again described using the reflected SDE formulation, based on the kinetic diagrams given above. The reflected SDEs for I_{Kr}, and I_{To1} are, respectively

$$dy_{Kr} = (\alpha_{X_r} - (\beta_{X_r} + \alpha_{X_r})y_{Kr})dt + \frac{1}{\sqrt{N_{Kr}}}\sqrt{\alpha_{X_r} + (\beta_{X_r} - \alpha_{X_r})y_{Kr}}dW_{Kr} + dK_{Kr}, \tag{3}$$

$$d\boldsymbol{y}_{To1} = H_{To1}\boldsymbol{y}_{To1}dt + \tfrac{1}{\sqrt{N_{To1}}}E_{To1}F_{To1}(\boldsymbol{y}_{To1})d\boldsymbol{W}_{To1} + d\boldsymbol{K}_{To1}, \tag{4}$$

where N_x is the number of channels of ion type x, $x = To1, Kr$, and the matrices H_{To1}, F_{To1}, E_{To1}, are given at the end of this section. In (3), y_{Kr} is simply the proportion of channels in the open state, and due to the conservation in channel numbers, the proportion of channels in the closed state is simply $1 - y_{Kr}$. The entries of the vectors $\boldsymbol{y}_{CaL}$, $\boldsymbol{y}_{Ks}$, and $\boldsymbol{y}_{To1}$ give the proportion of CaL, Ks and $To1$ channels, respectively, in each state, and the ordering of these vectors are defined at the end of this section. The most important state, in terms of the dynamics of the membrane potential, is the proportion of channels in the open state for each of these channels, which are as follows,

$$CaL^{open} = y_{CaL}(2) + y_{CaL}(4), \quad Ks^{open} = y_{Ks}(1) + y_{Ks}(2), \quad To1^{open} = y_{To1}(1). \tag{5}$$

At each time step the voltage and ionic concentrations are assumed to remain constant and the reflected SDEs are solved using the projection method described in [1]. The voltage and all other variables in the original Decker model are then updated using the Euler method. Due to the stiffness of this system, the time step needed to be taken is $< 0.009ms$ in order to resolve the upstroke of the AP. In the description of the matrices below the term $diag(\boldsymbol{v})$ describes a diagonal matrix (i.e. non-diagonal entries are 0) with diagonal entries given by the vector $\boldsymbol{v}$.

0.1 Matrices for I_{CaL} RSDE

$$\boldsymbol{y}_{CaL} = (C, O, C^{\star}, O^{\star}, CI, OI, CI^{\star}, OT^{\star})^T \, .$$

$$E_{CaL} = \begin{pmatrix} -1 & 0 & 0 & 0 & -1 & 0 & 0 & 0 & -1 & 0 & 0 & 0 \\ 1 & 0 & 0 & 0 & 0 & -1 & 0 & 0 & 0 & -1 & 0 & 0 \\ 0 & -1 & 0 & 0 & 1 & 0 & 0 & 0 & 0 & 0 & 1 & 0 \\ 0 & 1 & 0 & 0 & 0 & 1 & 0 & 0 & 0 & 0 & 0 & 1 \\ 0 & 0 & -1 & 0 & 0 & 0 & -1 & 0 & 1 & 0 & 0 & 0 \\ 0 & 0 & 1 & 0 & 0 & 0 & 0 & -1 & 0 & 1 & 0 & 0 \\ 0 & 0 & 0 & -1 & 0 & 0 & 1 & 0 & 0 & 0 & -1 & 0 \\ 0 & 0 & 0 & 1 & 0 & 0 & 0 & 1 & 0 & 0 & 0 & -1 \end{pmatrix} .$$

$$M_{CaL} = \begin{pmatrix} -(a+d+y) & b & e & 0 & x & 0 & 0 & 0 \\ a & -(b+d+y) & 0 & e & 0 & x & 0 & 0 \\ d & 0 & -(a+e+y^{\star}) & b & 0 & 0 & x^{\star} & 0 \\ 0 & d & a & -(b+e+y^{\star}) & 0 & 0 & 0 & x^{\star} \\ y & 0 & 0 & 0 & -(a+dI+x) & b & eI & 0 \\ 0 & y & 0 & 0 & a & -(b+x+dI) & 0 & eI \\ 0 & 0 & y^{\star} & 0 & dI & 0 & -(a+eI+x^{\star}) & b \\ 0 & 0 & 0 & y^{\star} & 0 & dI & a & -(b+eI+x^{\star}); \end{pmatrix} .$$

$$F_{CaL} = diag \begin{pmatrix} \sqrt{ay_{CaL,1}+by_{CaL,2}} \\ \sqrt{ay_{CaL,3}+by_{CaL,4}} \\ \sqrt{ay_{CaL,5}+by_{CaL,6}} \\ \sqrt{ay_{CaL,7}+by_{CaL,8}} \\ \sqrt{dy_{CaL,1}+ey_{CaL,3}} \\ \sqrt{dy_{CaL,2}+ey_{CaL,4}} \\ \sqrt{dIy_{CaL,5}+eIy_{CaL,7}} \\ \sqrt{dIy_{CaL,6}+eIy_{CaL,8}} \\ \sqrt{yy_{CaL,1}+xy_{CaL,5}} \\ \sqrt{yy_{CaL,2}+xy_{CaL,6}} \\ \sqrt{y^{\star}y_{CaL,3}+x^{\star}y_{CaL,7}} \\ \sqrt{y^{\star}y_{CaL,4}+x^{\star}y_{CaL,8};} \end{pmatrix} .$$

0.2 Matrices for I_{Ks} RSDE

$$\boldsymbol{y}_{Ks} = (O_2, O_1, C_{15}, C_{14}, C_{13}, C_{12}, C_{11}, C_{10}, C_9, C_8, C_7, C_6, C_5, C_4, C_3, C_2, C_1)^T \, .$$

$$E_{Ks} = \begin{pmatrix}
1 & 0 & 0 & 0 & 0 & 0 & 0 & 0 & 0 & 0 & 0 & 0 & 0 & 0 & 0 & 0 & 0 & 0 & 0 & 0 & 0 & 0 \\
-1 & 1 & 0 & 0 & 0 & 0 & 0 & 0 & 0 & 0 & 0 & 0 & 0 & 0 & 0 & 0 & 0 & 0 & 0 & 0 & 0 & 0 \\
0 & -1 & 1 & 0 & 0 & 0 & 0 & 0 & 0 & 0 & 0 & 0 & 0 & 0 & 0 & 0 & 0 & 0 & 0 & 0 & 0 & 0 \\
0 & 0 & -1 & 1 & 0 & 1 & 0 & 0 & 0 & 0 & 0 & 0 & 0 & 0 & 0 & 0 & 0 & 0 & 0 & 0 & 0 & 0 \\
0 & 0 & 0 & -1 & 1 & 0 & 0 & 0 & 0 & 0 & 0 & 0 & 0 & 0 & 0 & 0 & 0 & 0 & 0 & 0 & 0 & 0 \\
0 & 0 & 0 & 0 & 0 & -1 & 1 & 0 & 0 & 0 & -1 & 0 & 0 & 0 & 0 & 0 & 0 & 0 & 0 & 0 & 0 & 0 \\
0 & 0 & 0 & 0 & -1 & 0 & 1 & -1 & 0 & -1 & 0 & 0 & 0 & 0 & 0 & 0 & 0 & 0 & 0 & 0 & 0 & 0 \\
0 & 0 & 0 & 0 & 0 & 0 & 0 & 1 & -1 & 0 & 0 & 0 & 0 & 0 & 0 & 0 & 0 & 0 & 0 & 0 & 0 & 0 \\
0 & 0 & 0 & 0 & 0 & 0 & 0 & 0 & 0 & 0 & 1 & -1 & 0 & 0 & 0 & 0 & 0 & -1 & 0 & 0 & 0 & 0 \\
0 & 0 & 0 & 0 & 0 & 0 & 0 & 0 & 0 & 1 & 0 & 1 & -1 & 0 & 0 & 0 & -1 & 0 & 0 & 0 & 0 & 0 \\
0 & 0 & 0 & 0 & 0 & 0 & 0 & 0 & 1 & 0 & 0 & 0 & 1 & -1 & 0 & -1 & 0 & 0 & 0 & 0 & 0 & 0 \\
0 & 0 & 0 & 0 & 0 & 0 & 0 & 0 & 0 & 0 & 0 & 0 & 0 & 1 & -1 & 0 & 0 & 0 & 0 & 0 & 0 & 0 \\
0 & 0 & 0 & 0 & 0 & 0 & 0 & 0 & 0 & 0 & 0 & 0 & 0 & 0 & 0 & 0 & 0 & 1 & -1 & 0 & 0 & 0 \\
0 & 0 & 0 & 0 & 0 & 0 & 0 & 0 & 0 & 0 & 0 & 0 & 0 & 0 & 0 & 0 & 1 & 0 & 1 & -1 & 0 & 0 \\
0 & 0 & 0 & 0 & 0 & 0 & 0 & 0 & 0 & 0 & 0 & 0 & 0 & 0 & 0 & 1 & 0 & 0 & 0 & 1 & -1 & 0 \\
0 & 0 & 0 & 0 & 0 & 0 & 0 & 0 & 0 & 0 & 0 & 0 & 0 & 0 & 1 & 0 & 0 & 0 & 0 & 0 & 1 & -1 \\
0 & 0 & 0 & 0 & 0 & 0 & 0 & 0 & 0 & 0 & 0 & 0 & 0 & 0 & 0 & 0 & 0 & 0 & 0 & 0 & 0 & 1
\end{pmatrix}.$$

$$M_{Ks} = \begin{pmatrix}
-\delta_1 & p & 0 & 0 & 0 & 0 & 0 & 0 & 0 & 0 & 0 & 0 & 0 & 0 & 0 & 0 & 0 \\
w & -\delta_2 & u & 0 & 0 & 0 & 0 & 0 & 0 & 0 & 0 & 0 & 0 & 0 & 0 & 0 & 0 \\
0 & n & -\delta_3 & g & 0 & 0 & 0 & 0 & 0 & 0 & 0 & 0 & 0 & 0 & 0 & 0 & 0 \\
0 & 0 & 4d & -\delta_4 & a & 2g & 0 & 0 & 0 & 0 & 0 & 0 & 0 & 0 & 0 & 0 & 0 \\
0 & 0 & 0 & b & -\delta_5 & 0 & g & 0 & 0 & 0 & 0 & 0 & 0 & 0 & 0 & 0 & 0 \\
0 & 0 & 0 & 3d & 0 & -\delta_6 & a & 0 & 3g & 0 & 0 & 0 & 0 & 0 & 0 & 0 & 0 \\
0 & 0 & 0 & 0 & 3d & 2b & -\delta_7 & 2a & 0 & 2g & 0 & 0 & 0 & 0 & 0 & 0 & 0 \\
0 & 0 & 0 & 0 & 0 & 0 & b & -\delta_8 & 0 & 0 & g & 0 & 0 & 0 & 0 & 0 & 0 \\
0 & 0 & 0 & 0 & 0 & 2d & 0 & 0 & -\delta_9 & a & 0 & 0 & 4g & 0 & 0 & 0 & 0 \\
0 & 0 & 0 & 0 & 0 & 0 & 2d & 0 & 3b & -\delta_{10} & 2a & 0 & 0 & 3g & 0 & 0 & 0 \\
0 & 0 & 0 & 0 & 0 & 0 & 0 & 2d & 0 & 2b & -\delta_{11} & 3a & 0 & 0 & 2g & 0 & 0 \\
0 & 0 & 0 & 0 & 0 & 0 & 0 & 0 & 0 & 0 & b & -\delta_{12} & 0 & 0 & 0 & g & 0 \\
0 & 0 & 0 & 0 & 0 & 0 & 0 & 0 & d & 0 & 0 & 0 & -\delta_{13} & a & 0 & 0 & 0 \\
0 & 0 & 0 & 0 & 0 & 0 & 0 & 0 & 0 & d & 0 & 0 & 4b & -\delta_{14} & 2a & 0 & 0 \\
0 & 0 & 0 & 0 & 0 & 0 & 0 & 0 & 0 & 0 & d & 0 & 0 & 3b & -\delta_{15} & 3a & 0 \\
0 & 0 & 0 & 0 & 0 & 0 & 0 & 0 & 0 & 0 & 0 & d & 0 & 0 & 2b & -\delta_{16} & 4a \\
0 & 0 & 0 & 0 & 0 & 0 & 0 & 0 & 0 & 0 & 0 & 0 & 0 & 0 & 0 & b & -\delta_{17}
\end{pmatrix},$$

where the diagonal entries in the above matrix are given by,

$$(\delta_1, \ldots, \delta_4) = (w, (p+n), (u+4d), (g+3d+b)),$$

$$(\delta_5, \ldots, \delta_8) = ((a+3d), 2(g+d+b), (g+a+2d+b), 2(a+d)),$$

$$(\delta_9, \ldots, \delta_{12}) = ((3g+d+3b), (a+2g+d+2b), (2a+b+g+d), (3a+d)),$$

$$(\delta_{13}, \ldots, \delta_{17}) = (4(g+b), (a+3g+3b), 2(a+b+g), (b+3a+g), 4a).$$

$$F_{Ks} = diag \begin{pmatrix} \sqrt{wy_{Ks,1}+py_{Ks,2}} \\ \sqrt{uy_{Ks,3}+ny_{Ks,2}} \\ \sqrt{4dy_{Ks,3}+gy_{Ks,4}} \\ \sqrt{ay_{Ks,5}+by_{Ks,4}} \\ \sqrt{3dy_{Ks,5}+gy_{Ks,7}} \\ \sqrt{3dy_{Ks,4}+2gy_{Ks,6}} \\ \sqrt{ay_{Ks,7}+2by_{Ks,6}} \\ \sqrt{2ay_{Ks,8}+by_{Ks,7}} \\ \sqrt{2dy_{Ks,8}+gy_{Ks,11}} \\ \sqrt{2gy_{Ks,10}+2dy_{Ks,7}} \\ \sqrt{3gy_{Ks,9}+2dy_{Ks,6}} \\ \sqrt{ay_{Ks,10}+3by_{Ks,9}} \\ \sqrt{2ay_{Ks,11}+2by_{Ks,10}} \\ \sqrt{3ay_{Ks,12}+by_{Ks,11}} \\ \sqrt{dy_{Ks,12}+gy_{Ks,16}} \\ \sqrt{dy_{Ks,11}+2gy_{Ks,15}} \\ \sqrt{dy_{Ks,10}+3gy_{Ks,14}} \\ \sqrt{dy_{Ks,9}+4gy_{Ks,13}} \\ \sqrt{ay_{Ks,14}+4by_{Ks,13}} \\ \sqrt{2ay_{Ks,15}+3by_{Ks,14}} \\ \sqrt{3ay_{Ks,16}+2by_{Ks,15}} \\ \sqrt{4ay_{Ks,17}+by_{Ks,16}} \end{pmatrix}.$$

0.3 Matrices for I_{To1} RSDE

$$\boldsymbol{y}_{To1} = (y_{To1,1}, \ldots, y_{To1,16})^T , \tag{6}$$

where

$$(y_{To1,1}, \ldots, y_{To1,3}) = (\alpha_3 i_{f,1} i_{s,1}, \alpha_2 i_{f,1} i_{s,1}, \alpha_1 i_{f,1} i_{s,1}) ,$$

$$(y_{To1,4}, \ldots, y_{To1,6}) = (\alpha_0 i_{f,1}, i_{s,1}, \alpha_3 i_{f,0} i_{s,1}, \alpha_2 i_{f,0} i_{s,1}) ,$$

$$(y_{To1,7}, \ldots, y_{To1,9}) = (\alpha_1 i_{f,0} i_{s,1}, \alpha_0 i_{f,0} i_{s,1}, \alpha_3 i_{f,1} i_{s,0}) ,$$

$$(y_{To1,10}, \ldots, y_{To1,12}) = (\alpha_2 i_{f,1} i_{s,0}, \alpha_1 i_{f,1} i_{s,0}, \alpha_0 i_{f,1} i_{s,0}) ,$$

$$(y_{To1,13}, \ldots, y_{To1,16}) = (\alpha_3 i_{f,0} i_{s,0}, \alpha_2 i_{f,0} i_{s,0}, \alpha_1 i_{f,0} i_{s,0}, \alpha_0 i_{f,0} i_{s,0}) .$$

$$
E_{To1} = \begin{pmatrix}
1 & 0 & 0 & 0 & 0 & 0 & 0 & 0 & 0 & 0 & 0 & 0 & -1 & 0 & 0 & 0 & 0 & 0 & 0 & 0 & -1 & 0 & 0 & 0 & 0 & 0 & 0 & 0 \\
-1 & 1 & 0 & 0 & 0 & 0 & 0 & 0 & 0 & 0 & 0 & 0 & 0 & -1 & 0 & 0 & 0 & 0 & 0 & 0 & 0 & -1 & 0 & 0 & 0 & 0 & 0 & 0 \\
0 & -1 & 1 & 0 & 0 & 0 & 0 & 0 & 0 & 0 & 0 & 0 & 0 & 0 & -1 & 0 & 0 & 0 & 0 & 0 & 0 & 0 & -1 & 0 & 0 & 0 & 0 & 0 \\
0 & 0 & -1 & 0 & 0 & 0 & 0 & 0 & 0 & 0 & 0 & 0 & 0 & 0 & 0 & -1 & 0 & 0 & 0 & 0 & 0 & 0 & 0 & -1 & 0 & 0 & 0 & 0 \\
0 & 0 & 0 & 1 & 0 & 0 & 0 & 0 & 0 & 0 & 0 & 0 & 1 & 0 & 0 & 0 & 0 & 0 & 0 & 0 & 0 & 0 & 0 & 0 & -1 & 0 & 0 & 0 \\
0 & 0 & 0 & -1 & 1 & 0 & 0 & 0 & 0 & 0 & 0 & 0 & 0 & 1 & 0 & 0 & 0 & 0 & 0 & 0 & 0 & 0 & 0 & 0 & 0 & -1 & 0 & 0 \\
0 & 0 & 0 & 0 & -1 & 1 & 0 & 0 & 0 & 0 & 0 & 0 & 0 & 0 & 1 & 0 & 0 & 0 & 0 & 0 & 0 & 0 & 0 & 0 & 0 & 0 & -1 & 0 \\
0 & 0 & 0 & 0 & 0 & -1 & 0 & 0 & 0 & 0 & 0 & 0 & 0 & 0 & 0 & 1 & 0 & 0 & 0 & 0 & 0 & 0 & 0 & 0 & 0 & 0 & 0 & -1 \\
0 & 0 & 0 & 0 & 0 & 0 & 1 & 0 & 0 & 0 & 0 & 0 & 0 & 0 & 0 & -1 & 0 & 0 & 0 & 1 & 0 & 0 & 0 & 0 & 0 & 0 & 0 & 0 \\
0 & 0 & 0 & 0 & 0 & 0 & -1 & 1 & 0 & 0 & 0 & 0 & 0 & 0 & 0 & 0 & 0 & -1 & 0 & 0 & 0 & 1 & 0 & 0 & 0 & 0 & 0 & 0 \\
0 & 0 & 0 & 0 & 0 & 0 & 0 & -1 & 1 & 0 & 0 & 0 & 0 & 0 & 0 & 0 & 0 & -1 & 0 & 0 & 0 & 1 & 0 & 0 & 0 & 0 & 0 & 0 \\
0 & 0 & 0 & 0 & 0 & 0 & 0 & 0 & -1 & 0 & 0 & 0 & 0 & 0 & 0 & 0 & 0 & 0 & 0 & -1 & 0 & 0 & 0 & 1 & 0 & 0 & 0 & 0 \\
0 & 0 & 0 & 0 & 0 & 0 & 0 & 0 & 0 & 1 & 0 & 0 & 0 & 0 & 0 & 1 & 0 & 0 & 0 & 0 & 0 & 0 & 0 & 1 & 0 & 0 & 0 & 0 \\
0 & 0 & 0 & 0 & 0 & 0 & 0 & 0 & 0 & -1 & 1 & 0 & 0 & 0 & 0 & 0 & 0 & 1 & 0 & 0 & 0 & 0 & 0 & 0 & 0 & 1 & 0 & 0 \\
0 & 0 & 0 & 0 & 0 & 0 & 0 & 0 & 0 & 0 & -1 & 1 & 0 & 0 & 0 & 0 & 0 & 0 & 1 & 0 & 0 & 0 & 0 & 0 & 0 & 0 & 1 & 0 \\
0 & 0 & 0 & 0 & 0 & 0 & 0 & 0 & 0 & 0 & 0 & -1 & 0 & 0 & 0 & 0 & 0 & 0 & 0 & 1 & 0 & 0 & 0 & 0 & 0 & 0 & 0 & 1
\end{pmatrix}.
$$

$$
M_{To1} = \begin{pmatrix}
-d_1 & \alpha_a & 0 & 0 & \alpha_f & 0 & 0 & 0 & \alpha_s & 0 & 0 & 0 & 0 & 0 & 0 & 0 \\
3\beta_a & -d_2 & 2\alpha_a & 0 & 0 & \alpha_f & 0 & 0 & 0 & \alpha_s & 0 & 0 & 0 & 0 & 0 & 0 \\
0 & 2\beta_a & -d_3 & 3\alpha_a & 0 & 0 & \alpha_f & 0 & 0 & 0 & \alpha_s & 0 & 0 & 0 & 0 & 0 \\
0 & 0 & \beta_a & -d_4 & 0 & 0 & 0 & \alpha_f & 0 & 0 & 0 & \alpha_s & 0 & 0 & 0 & 0 \\
\beta_f & 0 & 0 & 0 & -d_5 & \alpha_a & 0 & 0 & 0 & 0 & 0 & 0 & \alpha_s & 0 & 0 & 0 \\
0 & \beta_f & 0 & 0 & 3\beta_a & -d_6 & 2\alpha_a & 0 & 0 & 0 & 0 & 0 & 0 & \alpha_s & 0 & 0 \\
0 & 0 & \beta_f & 0 & 0 & 2\beta_a & -d_7 & 0 & 0 & 0 & 0 & 0 & 0 & \alpha_s & 0 & 0 \\
0 & 0 & 0 & \beta_f & 0 & 0 & \beta_a & -d_8 & 0 & 0 & 0 & 0 & 0 & 0 & 0 & \alpha_s \\
\beta_s & 0 & 0 & 0 & 0 & 0 & 0 & 0 & -d_9 & 0 & 0 & \alpha_f & 0 & 0 & 0 & \\
0 & \beta_s & 0 & 0 & 0 & 0 & 0 & 0 & 3\beta_a & -d_{10} & 2\alpha_a & 0 & 0 & \alpha_f & 0 & 0 \\
0 & 0 & \beta_s & 0 & 0 & 0 & 0 & 0 & 0 & 2\beta_a & -d_{11} & 3\alpha_a & 0 & 0 & \alpha_f & 0 \\
0 & 0 & 0 & \beta_s & 0 & 0 & 0 & 0 & 0 & 0 & \beta_a & -d_{12} & 0 & 0 & 0 & \alpha_f \\
0 & 0 & 0 & 0 & \beta_s & 0 & 0 & 0 & \beta_f & 0 & 0 & 0 & -d_{13} & \alpha_a & 0 & 0 \\
0 & 0 & 0 & 0 & 0 & \beta_s & 0 & 0 & 0 & \beta_f & 0 & 0 & 3\beta_a & -d_{14} & 2\alpha_a & 0 \\
0 & 0 & 0 & 0 & 0 & 0 & \beta_s & 0 & 0 & 0 & \beta_f & 0 & 0 & 2\beta_a & -d_{15} & 3\alpha_a \\
0 & 0 & 0 & 0 & 0 & 0 & 0 & \beta_s & 0 & 0 & 0 & \beta_f & 0 & 0 & \beta_a & -d_{16}
\end{pmatrix},
$$

where the diagonal entries in the above matrix are,

$$(d_1, \dots, d_3) = (3\beta_a + \beta_f + \beta_s, \alpha_a + 2\beta_a + \beta_f + \beta_s, 2\alpha_a + \beta_a + \beta_f + \beta_s)\,,$$

$$(d_4, \dots, d_6) = (3\alpha_a + \beta_f + \beta_s, \alpha_f + 3\beta_a + \beta_s, \alpha_a + \alpha_f + 2\beta_a + \beta_s)\,,$$

$$(d_7, \dots, d_9) = (2\alpha_a + \alpha_f + \beta_a + \beta_s, 3\alpha_a + \alpha_f + \beta_s, \alpha_s + 3\beta_a + \beta_f)\,,$$

$$(d_{10}, \dots, d_{12}) = (\alpha_a + \alpha_s + 2\beta_a + \beta_f, 2\alpha_a + \alpha_s + \beta_a + \beta_f, 3\alpha_a + \alpha_s + \beta_f)\,,$$

$$(d_{13}, \dots, d_{16}) = (3\alpha_a + \alpha_s + \beta_f, \alpha_f + \alpha_s + 3\beta_a, \alpha_a + \alpha_f + \alpha_s + 2\beta_a, 2\alpha_a + \alpha_f + \alpha_s + \beta_a)\,.$$

$$F_{To1} = diag\begin{pmatrix} \sqrt{\alpha_a y_{To1,2}+3\beta_a y_{To1,1}} \\ \sqrt{2\alpha_a y_{To1,3}+2\beta_a y_{To1,2}} \\ \sqrt{3\alpha_a y_{To1,4}+\beta_a y_{To1,3}} \\ \sqrt{\alpha_a y_{To1,6}+3\beta_a y_{To1,5}} \\ \sqrt{2\alpha_a y_{To1,7}+2\beta_a y_{To1,6}} \\ \sqrt{3\alpha_a y_{To1,8}+\beta_a y_{To1,7}} \\ \sqrt{\alpha_a y_{To1,10}+3\beta_a y_{To1,9}} \\ \sqrt{2\alpha_a y_{To1,11}+2\beta_a y_{To1,10}} \\ \sqrt{3\alpha_a y_{To1,12}+\beta_a y_{To1,11}} \\ \sqrt{\alpha_a y_{To1,14}+3\beta_a y_{To1,13}} \\ \sqrt{2\alpha_a y_{To1,15}+2\beta_a y_{To1,14}} \\ \sqrt{3\alpha_a y_{To1,16}+\beta_a y_{To1,15}} \\ \sqrt{\beta_f y_{To1,1}+\alpha_f y_{To1,5}} \\ \sqrt{\beta_f y_{To1,2}+\alpha_f y_{To1,6}} \\ \sqrt{\beta_f y_{To1,3}+\alpha_f y_{To1,7}} \\ \sqrt{\beta_f y_{To1,4}+\alpha_f y_{To1,8}} \\ \sqrt{\beta_f y_{To1,9}+\alpha_f y_{To1,13}} \\ \sqrt{\beta_f y_{To1,10}+\alpha_f y_{To1,14}} \\ \sqrt{\beta_f y_{To1,11}+\alpha_f y_{To1,15}} \\ \sqrt{\beta_f y_{To1,12}+\alpha_f y_{To1,16}} \\ \sqrt{\beta_s y_{To1,1}+\alpha_s y_{To1,9}} \\ \sqrt{\beta_s y_{To1,2}+\alpha_s y_{To1,10}} \\ \sqrt{\beta_s y_{To1,3}+\alpha_s y_{To1,11}} \\ \sqrt{\beta_s y_{To1,4}+\alpha_s y_{To1,12}} \\ \sqrt{\beta_s y_{To1,5}+\alpha_s y_{To1,13}} \\ \sqrt{\beta_s y_{To1,6}+\alpha_s y_{To1,14}} \\ \sqrt{\beta_s y_{To1,7}+\alpha_s y_{To1,15}} \\ \sqrt{\beta_s y_{To1,8}+\alpha_s y_{To1,16}} \end{pmatrix}.$$

References

[1] Dangerfeild CE, Kay D, Burrage K (2012) Modeling ion channel dynamics through reflected stochastic differential equations. *Phys Rev E*, **85**

[2] Goldwyn JH, Imennov NS, Famulare M, Shea-Brown E, Stochastic differential equation models for ion channel noise in Hodgkin-Huxley neurons. *Phys Rev E*, **83**
